# The causal effects of inflammatory bowel disease on its ocular manifestations: A Mendelian randomization study

**DOI:** 10.1371/journal.pone.0316437

**Published:** 2025-03-12

**Authors:** Lian Luo, Xiaowei Tang, Jia Xu, Yuxi Bao, Xinyue Hu, Xiaolin Zhong

**Affiliations:** 1 Department of Gastroenterology, The Affiliated Hospital of Southwest Medical University, Luzhou, China; 2 Nuclear Medicine and Molecular Imaging Key Laboratory of Sichuan Province, Luzhou, China; University College London, UNITED KINGDOM OF GREAT BRITAIN AND NORTHERN IRELAND

## Abstract

**Background:**

Observational studies have shown that ocular manifestations of inflammatory bowel disease (IBD) are common extraintinal manifestations, among which iridocyclitis, scleritis and episcleritis are the most common. However, whether there is a causal relationship between the two is unclear. The purpose of this study was to evaluate the causality of IBD on ocular manifestations using the mendelian randomization (MR) analysis.

**Methods:**

We performed a two-sample MR analysis with public genome-wide association studies (GWAS) data. Eligible instrumental variables (IVs) were selected according to the three assumptions of MR analysis. The inverse-variance weighted (IVW) method was the main method. Complementary methods included the MR-Egger regression, the Weighted Median, the Weighted Mode and MR pleiotropy residual sum and outlier (MR-PRESSO) methods.

**Results:**

After false discovery rate (FDR) correction, genetically predicted IBD (IVW OR =  1.184, 95% CI: 1.125-1.247, P_FDR <  0.001), Crohn’s disease (CD, IVW OR =  1.082, 95% CI: 1.033-1.133, P_FDR =  0.007) and ulcerative colitis (UC, IVW OR =  1.192, 95% CI: 1.114-1.275, P_FDR <  0.001) were associated with an increased risk of iridocyclitis. Moreover, IBD (IVW OR =  1.128, 95% CI: 1.064-1.196, P_FDR =  0.001), CD (IVW OR =  1.077, 95% CI: 1.026-1.131, P_FDR =  0.019) and UC (IVW OR =  1.153, 95% CI: 1.069-1.243, P_FDR = 0.003) were associated with a higher risk of uveitis (uveitis includes iridocyclitis). Further sensitivity analyses validated the robustness of the above associations. However, IBD and its subtypes were not associated with scleritis, episcleritis, optic neuritis and corneal disease. Results of complementary methods were generally consistent with those of the IVW method.

**Conclusions:**

Our study revealed genetically predicted associations of IBD, CD and UC on iridocyclitis and uveitis in European populations. However, IBD, CD, and UC are not causally related to scleritis, external scleritis, optic neuritis, and corneal disease.

## Introduction

Inflammatory bowel disease (IBD), comprising Crohn’s disease (CD) and ulcerative colitis (UC), is a global disease, and the incidence of which is increasing year by year. Its etiology remains elusive and intricate, posing a huge challenge in modern medicine. IBD usually coexists with extraintestinal manifestations (EIMs), including musculoskeletal, ocular, skin, hepatobiliary, pulmonary, and endocrine involvement [1]. Ocular manifestations are common EIMs, contributing significantly to the health burden. Episcleritis, scleritis, and iridocyclitis are the most prevalent ocular manifestations of IBD. Additional reported ocular manifestations include corneal disease, retinal vasculitis, papillitis, corneal infiltrates, myositis, scleromalacia perforans, and optic neuritis [2]. Although the association between IBD and ocular manifestations has long intrigued researchers, the exact nature of this relationship has still not been elucidated.

Conventional epidemiological approaches often face limitations, such as confounding factors and reverse causation, which hinder the exploration of the causal relationship between IBD and ocular manifestations. Mendelian randomisation (MR) is a novel epidemiological approach that estimates the causal relationship between exposure and outcome using genetic variants as instrumental variables (IVs) [3]. By exploiting the random allocation of alleles during gametogenesis, MR avoids confounding and reverse causality in observational studies, providing a innovative and robust method for investigating causality [4].

In recent years, MR has become a powerful tool for exploring interactions among various diseases and traits. Additionally, genome-wide association studies (GWAS) have identified numerous genetic loci associated with IBD susceptibility, providing a rich source of IVs for MR studies and enabling researchers to more precisely explore the causal relationship between IBD and ocular manifestations. This study aims to provide insights into the causal relationship between IBD and ocular manifestations by synthesizing MR results, suggesting new avenues for research and clinical practice, and highlighting the importance of ophthalmic examinations in IBD patients.

## Methods

We conducted a MR analysis to investigate the potential causal relationships between IBD and its ocular manifestations. Multiple single nucleotide polymorphisms (SNPs) representing genetic variation were selected as IVs for MR analysis. Three key assumptions were adopted: (1) IVs are directly related to the exposure; (2) IVs are independent of any confounding variables; (3) IVs are not directly related to the outcome [5].

### Data source

Data was extraceted from GWAS database, including GWAS Catalog and Finngen. Since all data used were already published in the public databases, no additional ethical approval was required. All cohort populations were of European descent to reduce the bias due to ethnic differences. We obtained summary statistics for genetic associations with IBD from large-scale GWAS conducted by the International IBD Genetics Consortium (IIBDGC). Diagnosis of IBD and its subtypes were determined by the combination of endoscopic, radiological, and histopathological criteria. Database information on IBD and its subtypes as well as ocular manifestations is provided in Table 1.

**Table 1:** Details of the studies included in the Mendelian randomization analyses.

Phenotype	ncase	ncontrol	Sample size	Number of SNPs	Year	Web source
IBD	31,665	33,977	65,642	157,116	2015	https://gwas.mrcieu.ac.uk/
CD	17,897	33,977	51,874	124,888	2015	https://gwas.mrcieu.ac.uk/
UC	13,768	33,977	47,745	156,116	2015	https://gwas.mrcieu.ac.uk/
Iridocyclitis	3,622	209,287	212,909	16,380,395	2021	https://www.finngen.fi/en
Uveitis	2,616	478,126	480,742	24,194,599	2021	https://gwas.mrcieu.ac.uk/
Scleritis	121	209,287	209,408	16,380,407	2021	https://www.finngen.fi/en
Episcleritis	660	209,287	209,947	16,380,407	2021	https://www.finngen.fi/en
Optic neuritis	582	217,491	218,073	16,380,463	2021	https://www.finngen.fi/en
Corneal disease	124	456,224	456,348	11,831,932	2021	https://gwas.mrcieu.ac.uk/

IBD, inflammatory bowel disease; CD, Crohn’s disease; UC, ulcerative colitis; SNPs, single-nucleotide polymorphisms.

### SNP selection

In this study, a genome-wide significance level of p <  1 × 10^ − 8^ was utilized to identify SNPs associated with exposure, and a clustering algorithm with a cutoff of r^2^ =  0.001 and kb =  10,000 was employed to avoid linkage disequilibrium (LD). The strength of the IVs was then assessed using an F statistic greater than 10 to minimize the impact of weak IVs on the causal analysis. Through harmonization, ambiguous and palindromic SNPs were eliminated. To mitigate potential pleiotropy, PhenoScanner V2 (http://www.phenoscanner.medschl.cam.ac.uk/) was used to exclude IVs associated with confounding factors [6].

### MR analyses

R software 4.3.1 was used in this study. The primary MR analysis method is inverse variance weighting (IVW), which derives an overall estimate of the exposure’s effect on the outcome by aggregating Wald estimates of causality for each IV [7]. Additionally, MR-Egger regression, weighted median, weighted mode, and MR-PRESSO tests were supplemented to estimate the causal relationship and detect potential pleiotropic effects [8,9]. Results are reported as odds ratios (ORs) and 95% confidence intervals (CIs), with p-values <  0.05 considered statistically significant. The statistical significance of the MR effect estimates was defined as a false discovery rate (FDR) of < 5% to adjust for multiple testing.

### Sensitivity analysis

**Fig 1:**
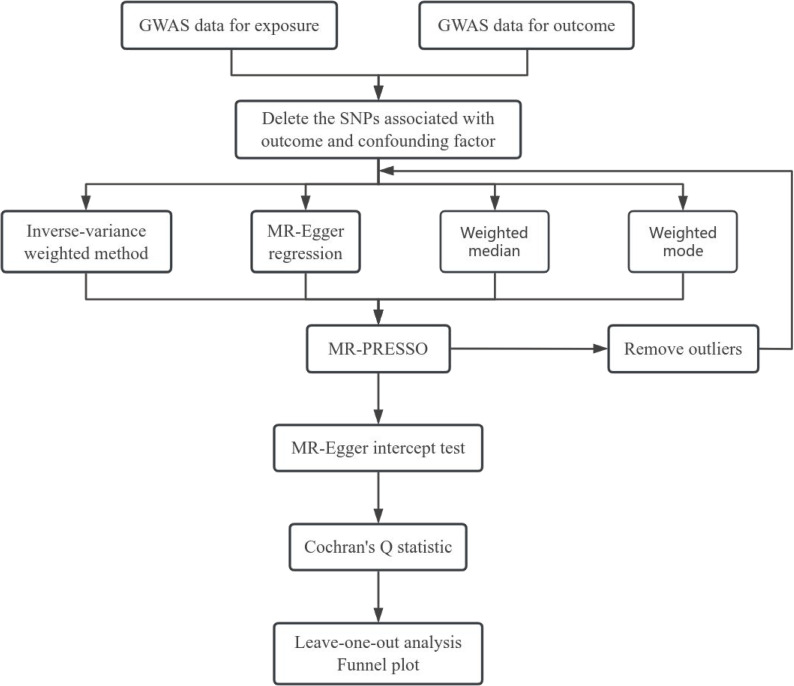
Flow chart of the study.

To evaluate the presence of horizontal pleiotropy, we employed the MR-Egger intercept test, where a significant intercept (P <  0.05) indicates pleiotropy, warranting cautious interpretation of the results. The outcomes of the MR-Egger intercept test were depicted using scatter plots[9]. Additionally, the MR-PRESSO outlier test was used to identify and exclude SNPs with significant differences, thus correcting for horizontal pleiotropy [10]. Cochran’s Q statistic was utilized to assess heterogeneity among the included IVs, with P <  0.05 indicating significant heterogeneity [11]. The results were visualized using funnel plots. Furthermore, we conducted leave-one-out sensitivity tests by removing each IV in turn and performing MR analysis on the remaining IVs. This allowed us to determine whether abnormal IVs significantly influenced causal effect estimates and to evaluate the stability of the effect estimates [12]. The main study procedures was visualized in Fig 1.

## Result

### SNPs selected in MR

We identified 123 SNPs for IBD, 118 SNPs for CD, and 68 SNPs for UC that met the widely accepted genome-wide significance threshold (P <  1 ×  10^–8^, r^2^ <  0.001, kb =  10,000) for exposure. Ambiguous and palindromic SNPs, as well as outliers identified in the MR-PRESSO analysis, were removed. Finally, by using PhenoScanner V2, among the SNPs included in the analysis, no SNPs were found to be associated with an outcome related phenotype, such as smoking [13]. The general information of the chosen SNPs was listed in S1-S18 Tables.

### MR analysis

**Fig 2:**
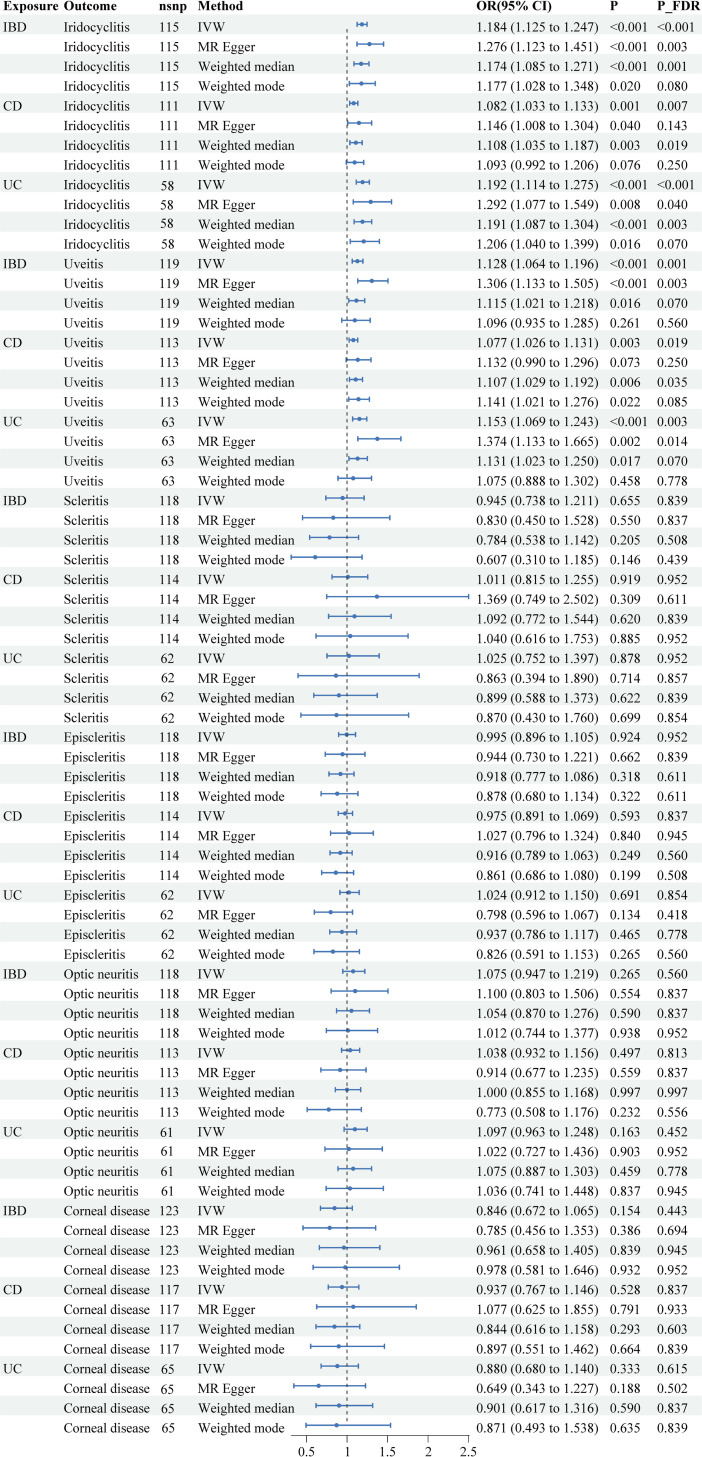
Forest plots of MR analysis on the causal relationship between exposures and outcomes.

Among the common ocular manifestations of IBD, after FDR correction, IVW analysis indicated a significant positive correlation between IBD (IVW OR =  1.184, 95% CI: 1.125-1.247, P_FDR <  0.001), CD (IVW OR =  1.082, 95% CI: 1.033-1.133, P_FDR =  0.007), UC (IVW OR =  1.192, 95% CI: 1.114-1.275, P_FDR <  0.001), and iridocyclitis. Moreover, IBD (IVW OR =  1.128, 95% CI: 1.064-1.196, P_FDR =  0.001), CD (IVW OR =  1.077, 95% CI: 1.026-1.131, P_FDR =  0.019), and UC (IVW OR =  1.153, 95% CI: 1.069-1.243, P_FDR =  0.003) are associated with a higher risk of uveitis, which includes iridocyclitis. The results of complementary methods were generally consistent with those obtained using the IVW method. However, non-statistically significiant causal relationship was observed between IBD, CD, UC and scleritis (IVW P_FDR_IBD-scleritis_ =  0.839, P_FDR_CD-scleritis_ =  0.952, P_FDR_UC-scleritis_ =  0.952) or episcleritis (IVW P_FDR_IBD-episcleritis_ =  0.952, P_FDR_CD-episcleritis_ =  0.837, P_FDR_UC-episcleritis_ =  0.854). Similarly, IBD and its subtypes have not been found to have a causal relationship with optic neuritis (IVW P_FDR_IBD-optic neuritis_ =  0.560, P_FDR_CD-optic neuritis_ =  0.813, P_FDR_UC-optic neuritis_ =  0.452) or corneal diseases (IVW P_FDR_IBD-corneal diseases_ =  0.442, P_FDR_CD-corneal diseases_ =  0.837, P_FDR_UC-corneal diseases_ =  0.615). The MR results are presented in Table 2, Fig 2 and S19 Table.

**Table 2:** Results of Mendelian analysis between exposures and outcomes using inverse-variance weighted method.

Exposure	Outcome	Method	nsnp	b	se	or	P	P_FDR
IBD	Iridocyclitis	IVW	115	0.169	0.026	1.184	<0.001	<0.001
CD		IVW	111	0.078	0.024	1.082	0.001	0.007
UC		IVW	58	0.175	0.034	1.192	<0.001	<0.001
IBD	Uveitis	IVW	119	0.120	0.030	1.128	<0.001	0.001
CD		IVW	113	0.074	0.025	1.077	0.003	0.019
UC		IVW	63	0.142	0.039	1.153	<0.001	0.003
IBD	Scleritis	IVW	118	-0.057	0.126	0.945	0.655	0.839
CD		IVW	114	0.011	0.110	1.011	0.919	0.952
UC		IVW	62	0.024	0.158	1.025	0.878	0.952
IBD	Episcleritis	IVW	118	-0.005	0.053	0.995	0.924	0.952
CD		IVW	114	-0.025	0.046	0.975	0.593	0.837
UC		IVW	62	0.024	0.059	1.024	0.691	0.854
IBD	Optic neuritis	IVW	118	0.072	0.065	1.075	0.265	0.560
CD		IVW	113	0.037	0.055	1.038	0.497	0.813
UC		IVW	61	0.092	0.066	1.097	0.163	0.452
IBD	Corneal disease	IVW	123	-0.168	0.118	0.846	0.154	0.443
CD		IVW	117	-0.065	0.102	0.937	0.528	0.837
UC		IVW	65	-0.128	0.132	0.880	0.333	0.615

IBD, inflammatory bowel disease; CD, Crohn’s disease; UC, ulcerative colitis; snp, single-nucleotide polymorphism; IVW, inverse-variance weighted; or, odds ratio; FDR, false discovery rate.

### Sensitivity analysis

**Fig 3:**
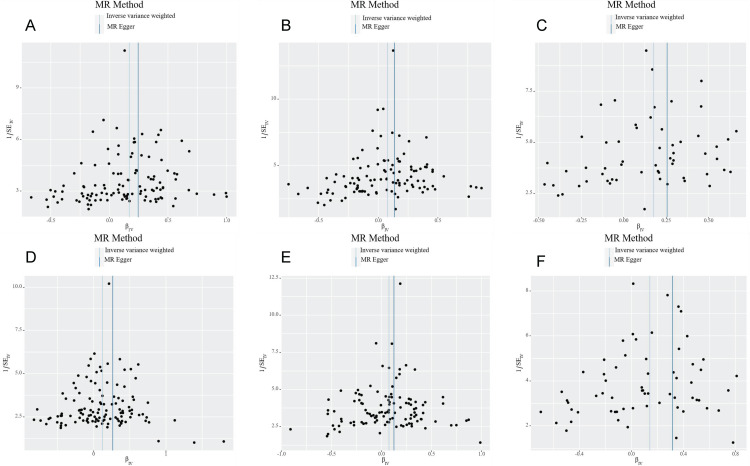
Funnel plots of heterogeneity analysis on the causal relationship between exposures and outcomes. (A) IBD and iridocyclitis, (B) CD and iridocyclitis, (C) UC and iridocyclitis, (D) IBD and uveitis, (E) CD and uveitis, (F) UC and uveitis.

**Fig 4:**
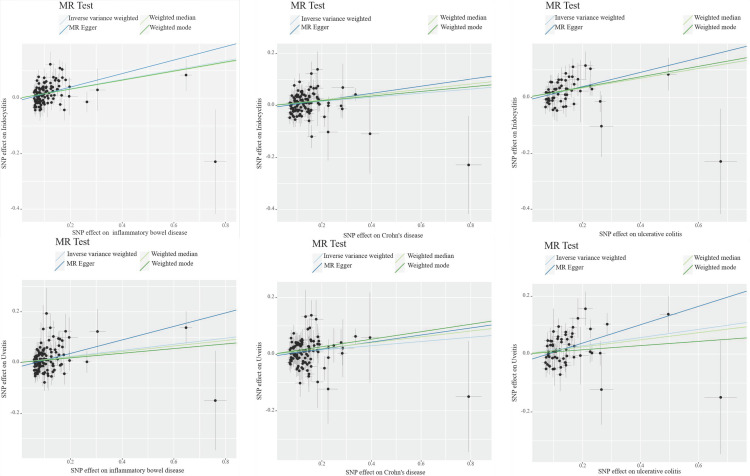
Scatter plots of the MR analysis. The slope of each line represents the effect estimated by an MR method.

**Fig 5:**
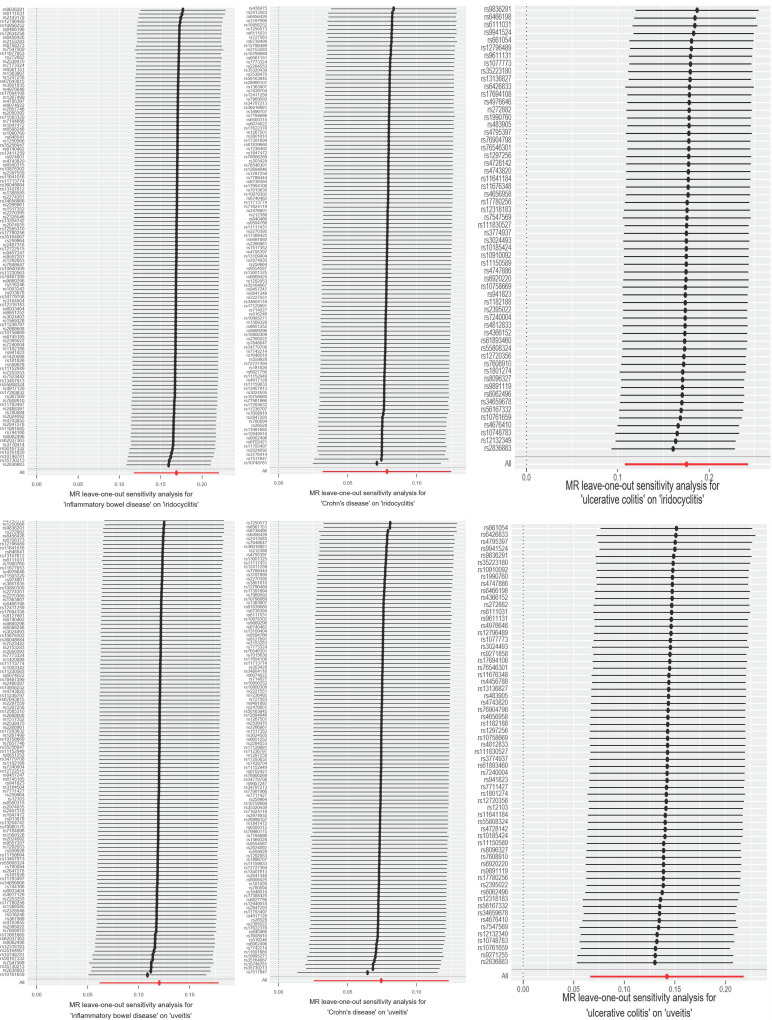
The Forest plot of leave-one-out sensitivity analysis. This figure showing the impact of each SNP on the overall causal estimate to outcome.

To assess the robustness of MR analysis, we conducted a series of sensitivity analysis, including cochran Q test, MR Egger intercept test and MR - PRESSO test. The Q test analysis revealed heterogeneity between CD, UC and iridocyclitis, as well as between UC and uveitis, with no observed heterogeneity in other outcomes. Funnel plots shown in Fig 3 and S1 Fig. The use of random-effects IVW as the primary estimation method adequately accounted for acceptable heterogeneity [3]. Additionally, MR-Egger intercept test results showed P values greater than 0.05 for all outcomes except IBD (MR Egger intercept =  -0.017, P =  0.029) and uveitis, suggesting no significant pleiotropic bias for the remaining outcomes (Table 3, Fig 4 and S2 Fig). Furthermore, leave-one-out analysis indicated that no SNP had a significant influence on the results (Fig 5 and S3 Fig), indicating no violations in estimation. The MR-PRESSO test identified horizontal pleiotropy between CD, UC and iridocyclitis, as well as between UC and uveitis. The results of sensitivity analyses for the other outcomes were shown in Supplementary Figure.

**Table 3:** Sensitivity analysis of the causal association between exposures and the risk of outcomes.

Exposure	Outcome	Cochran’s Q value	Q test P	MR-egger intercept	MR-egger intercept P	MR-PRESSO global test P
IBD	Iridocyclitis	134.291	0.094	-0.009	0.212	0.098
CD		141.489	0.023	-0.008	0.344	0.024
UC		87.246	0.006	-0.012	0.353	0.007
IBD	Uveitis	141.344	0.070	-0.017	0.029	0.075
CD		126.570	0.164	-0.007	0.437	0.157
UC		99.180	0.002	-0.026	0.057	0.002
IBD	Scleritis	121.032	0.381	0.016	0.648	0.384
CD		117.609	0.364	-0.042	0.294	0.361
UC		80.179	0.050	0.026	0.642	0.054
IBD	Episcleritis	101.762	0.841	0.006	0.663	0.840
CD		99.388	0.816	-0.007	0.674	0.823
UC		59.131	0.544	0.038	0.072	0.536
IBD	Optic neuritis	147.163	0.031	-0.003	0.874	0.023
CD		134.566	0.072	0.018	0.376	0.059
UC		60.539	0.456	0.010	0.660	0.466
IBD	Corneal disease	101.358	0.913	0.009	0.769	0.911
CD		104.582	0.768	-0.020	0.593	0.773
UC		58.003	0.687	0.046	0.309	0.717

IBD, inflammatory bowel disease; CD, Crohn’s disease; UC, ulcerative colitis; MR-PRESSO, MR pleiotropy residual sum and outlier.

## Discussion

This study investigated the causal relationship between IBD and its primary ocular manifestations using MR analysis with SNPs as IVs. The results showed that genetic predictors of IBD, including UC and CD, were associated with an elevated risk of iridocyclitis and uveitis. Acuminate granulomatous anterior uveitis, affecting the iris and ciliary body, was identified as the most common type of uveitis in IBD. Middle, posterior, or panuveitis can also coexist with IBD but less commonly. Furthermore, there was no evidence supporting an association between genetic predictors of IBD and an increased risk of scleritis, episcleritis, corneal disease, or optic neuritis. The observed heterogeneity in this study was deemed acceptable, and the robustness of most findings was confirmed by conducting various sensitivity analyses.

Studies indicate that ocular manifestations affect 2-6% of patients with IBD, with CD and female gender independently linked to higher risks of such manifestations [14,15]. However, the study by Roberts et al. found that ocular EIMs were significantly more common in smokers compared to nonsmokers in UC, but not in CD [13]. Additionally, exposure to respirable silica dust has been associated with an increased risk of uveitis [16].

The mechanisms underlying the ocular manifestations of IBD are complex and uncertain, involving interactions among immune system imbalance, genetic predisposition, environmental factors, and alterations in the gut microbiota. The development of ocular manifestations in IBD may involve the formation of circulating antigen-antibody complexes. Additionally, it may involve the production of autoantibodies targeting antigens shared between the colon and other tissues, including the eye [17]. For instance, peptide 7E12H12 is found in the colonic epithelium and nonpigmented ciliary epithelial cells [18]. In all EIMs, ocular and cutaneous symptoms typically co-occur with other EIMs, with ocular manifestations and erythema nodosum showing a high likelihood of coexistence. Evidence suggests a shared antigen in the ciliary epithelium of the eye, chondrocytes, and intestine, potentially contributing to the triad of EIMs—retinitis, arthritis, and erythema nodosum—in some IBD patients [19]. Autoreactive T cells and their various cytokines, including IL-6, IL-10, and IL-17, have been implicated in the pathogenesis of ocular EIMs and IBD, suggesting a possible common immune pathogenesis [20–24]. Greater intestinal damage and permeability caused by transmural inflammation in CD may explain the higher incidence of ocular manifestations compared to UC [21].

Second, there appears to be a genetic predisposition to the pathogenesis of EIMs. A positive family history of IBD is linked to persistent EIMs in patients with CD, and may independently increase the risk of ocular inflammation [25]. Specifically, the onset of ocular EIMs has been linked to variants in the gene loci encoding HLA-B27, HLA-B58 and HLA-DRB1 * 0103 [26]. Moreover, studies have found that vitamin D is negatively correlated with the development of several autoimmune diseases, including CD, UC and iridocyclitis, and it has an impact on both innate and adaptive immune responses [27,28].

Macrophage-mediated autophagy could represent another pathogenic mechanism. Santeford et al. discovered that a specific polymorphism (Thr300Ala or T300A) in the autophagy gene ATG16L1 is linked to an elevated risk of developing CD and uveitis [29]. The gut microbiota and biological dysregulation may contribute to the pathogenesis of ocular EIMs through molecular mimicry. In a mouse model of idiopathic uveitis, Horai et al. demonstrated that the activation of retina-specific T cells depends on symbiotic gut microbes. This activation occurs through signaling via autoreactive T-cell receptors (TCRs) in response to non-homologous antigens in the gut, independent of endogenous retinal autoantigens [30].

The presence of uveitis in IBD patients may not necessarily correlate with the disease activity of IBD, possibly occurring even before the diagnosis of IBD, and it is associated with an aggressive phenotype and a more severe disease course [31,32]. Patients with uveitis are at a heightened risk of developing abscesses, fistulas, and other EIMs. Adalimumab is the first and only non-corticosteroid drug approved by the US Food and Drug Administration (FDA) for treating non-infectious intermediate, posterior, and panuveitis in adults [33]. For anterior uveitis, initial treatment typically involves topical steroids and/or cyclophosphamide, sometimes with systemic corticosteroids. If uveitis is severe or persistent, or if there is inflammation affecting other parts of the eye, whole-body immunosuppressive therapy may be necessary. Drugs targeting tumor necrosis factor-α(TNF-α) are considered first-line immunomodulatory therapy for uveitis, with antimetabolites more commonly used as adjuvant or second-line therapy [34].

Episcleritis is closely associated with outbreaks of underlying IBD and requires effective control of mucosal disease activity. Initial treatment includes cold compresses or the use of topical lubricants [35]. If symptoms persist, topical nonsteroidal antiinflammatory drugs (NSAIDs) or corticosteroid eye drops may be used, although NSAIDs may exacerbate IBD flares [36]. Unlike episcleritis, scleritis is typically not associated with IBD outbreaks in IBD patients. First-line treatment typically involves oral NSAIDs, with steroids and immunosuppressive agents also used for scleritis. Refractory scleritis may require treatment with biologic agents, such as adalimumab and infliximab [37].

The incidence of keratopathy is higher in IBD patients compared to age-matched healthy controls [38]. Mild keratopathy can initially be treated with over-the-counter artificial tears. Excluding infection, more severe keratopathy may require treatment with topical corticosteroids, despite the risk of corneal thinning [31,39].

Many other ocular manifestations in IBD patients are associated with medication use. Cataracts, central serous retinopathy, optic neuropathy secondary to idiopathic intracranial hypertension, glaucoma, retinopathy, and others may occur with long-term steroid use [40]. Additionally, adalimumab has been associated with corneal immune infiltration and diffuse retinopathy [41]. Optic neuropathy and retinal vein thrombosis have been reported with infliximab and cyclosporine use.

There are several limitations of this study that need to be acknowledged. Firstly, the data used in the study were derived from a European population. Genetic and environmental factors may vary across populations, potentially limiting the generalizability of the findings to other continental populations. Secondly, a small number of studies indicated potential horizontal pleiotropy, possibly influenced by confounding factors (such as ocular manifestations related to drug use), affecting the accuracy of the results. Additionally, due to the small sample size, it was difficalut to investigate the association between each corneal disease and IBD separately. Fourthly, the absence of GWAS databases for some eye diseases results in incomplete studies.

## Conclusions

This study utilized GWAS data to perform MR analyses investigating potential causal relationships between IBD and ocular manifestations. The results confirm that genetic predictors of IBD significantly influence the development of iridocyclitis and uveitis. However, there was no compelling evidence linking genetic predictors of IBD to other ocular manifestations. Ocular manifestations are common EIMs. During diagnosis, treatment, and follow-up, screening for ocular manifestations is crucial in high-risk groups, particularly for iridocyclitis.

## Supporting information

S1 Fig Funnel plots of the MR analysis. (A) IBD and scleritis, (B) CD and scleritis, (C) UC and scleritis, (D) IBD and episcleritis, (E) CD and episcleritis, (F) UC and episcleritis, (G) IBD and optic neuritis, (H) CD and optic neuritis, (I) UC and optic neuritis, (J) IBD and corneal disease, (K) CD and corneal disease, (L) UC and corneal disease.
(PDF)

S2 Fig Scatter plots of the MR analysis. (PDF)

S3 Fig The Forest plot of leave-one-out sensitivity analysis. (PDF)

S1 Table The general information of the chosen genetic IVs for IBD on iridocyclitis. (DOC)

S2 Table The general information of the chosen genetic IVs for CD on iridocyclitis. (DOC)

S3 Table The general information of the chosen genetic IVs for UC on iridocyclitis. (DOC)

S4 Table The general information of the chosen genetic IVs for IBD on uveitis. (DOC)

S5 Table The general information of the chosen genetic IVs for CD on uveitis. (DOC)

S6 Table The general information of the chosen genetic IVs for UC on uveitis. (DOC)

S7 Table The general information of the chosen genetic IVs for IBD on scleritis. (DOC)

S8 Table The general information of the chosen genetic IVs for CD on scleritis. (DOC)

S9 Table The general information of the chosen genetic IVs for UC on scleritis. (DOC)

S10 Table The general information of the chosen genetic IVs for IBD on episcleritis. (DOC)

S11 Table The general information of the chosen genetic IVs for CD on episcleritis. (DOC)

S12 Table The general information of the chosen genetic IVs for UC on episcleritis. (DOC)

S13 Table The general information of the chosen genetic IVs for IBD on optic neuritis. (DOC)

S14 Table The general information of the chosen genetic IVs for CD on optic neuritis. (DOC)

S15 Table The general information of the chosen genetic IVs for UC on optic neuritis. (DOC)

S16 Table The general information of the chosen genetic IVs for IBD on corneal disease. (DOC)

S17 Table The general information of the chosen genetic IVs for CD on corneal disease. (DOC)

S18 Table The general information of the chosen genetic IVs for UC on corneal disease. (DOC)

S19 Table Results of MR analyses. (DOC)
