# Supplementary figures and images for: The causal effects of inflammatory bowel disease on its ocular manifestations: A Mendelian randomization study
Source: PLoS One. 2025 Mar 12;20(3):e0316437. doi: 10.1371/journal.pone.0316437 (PMC11902285; doi:10.1371/journal.pone.0316437)

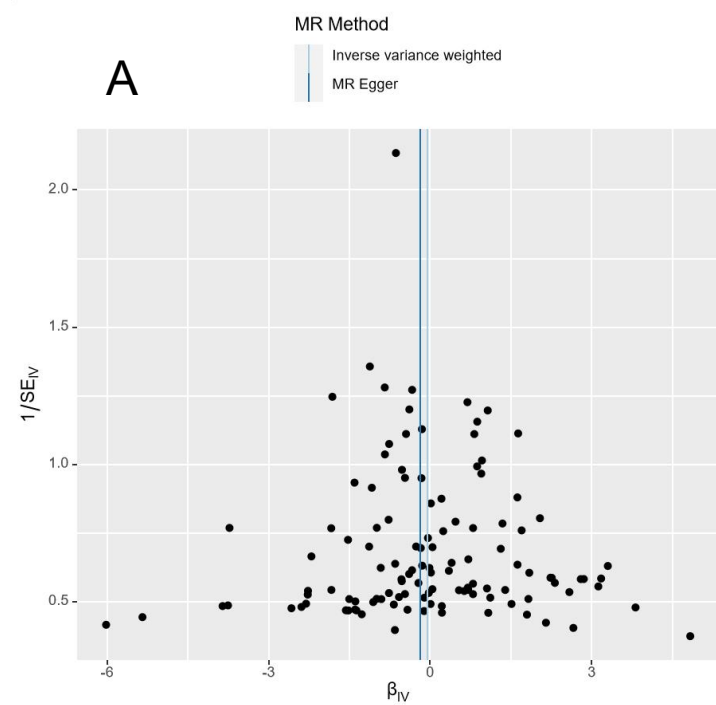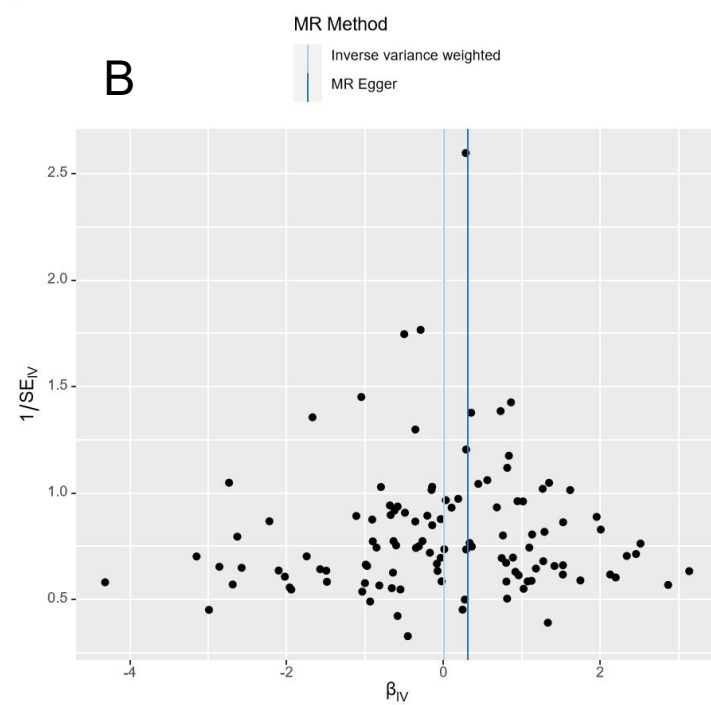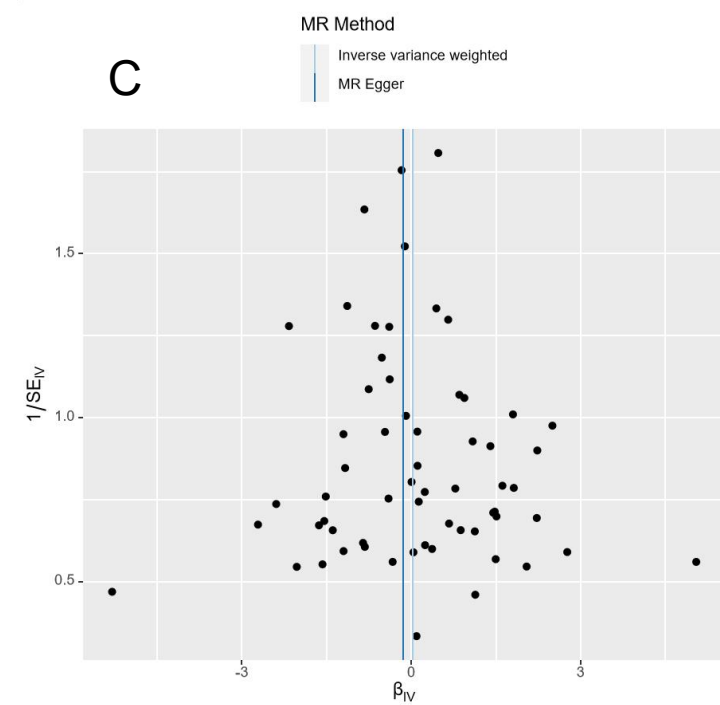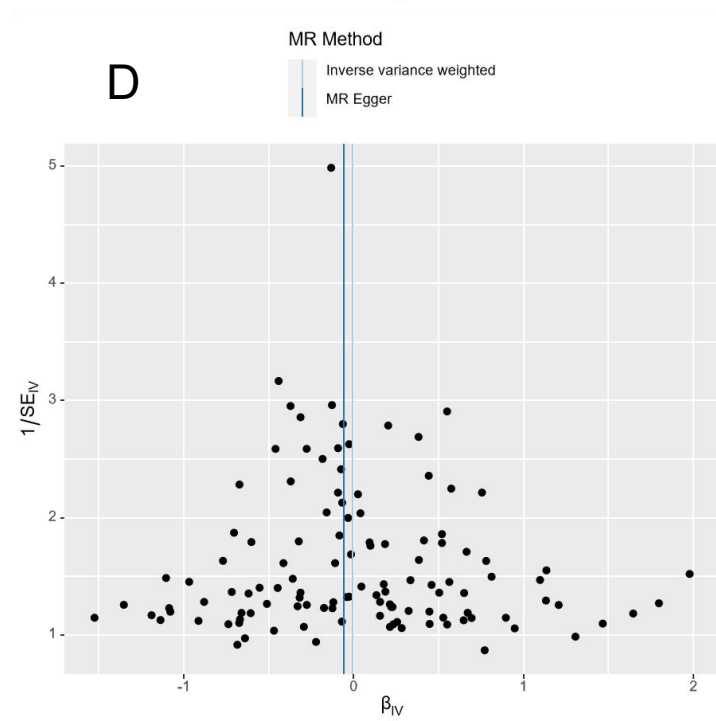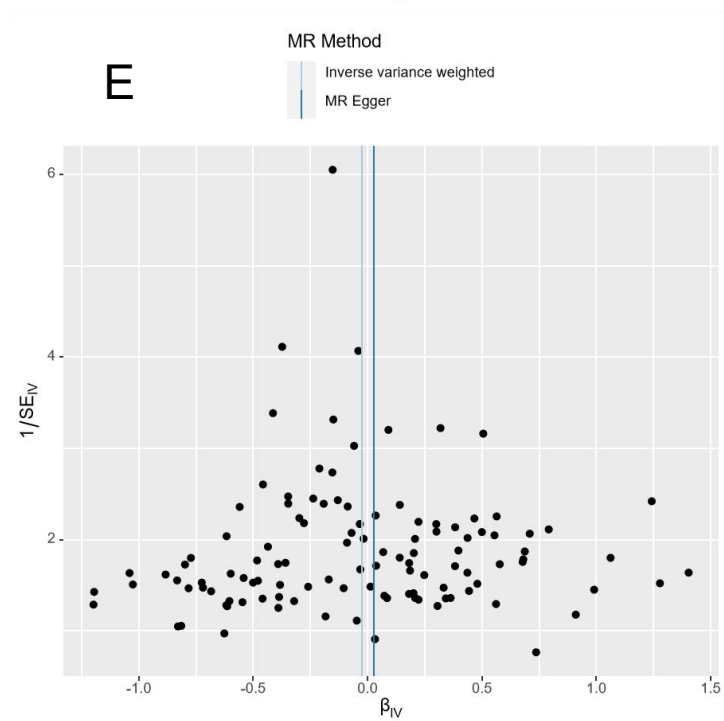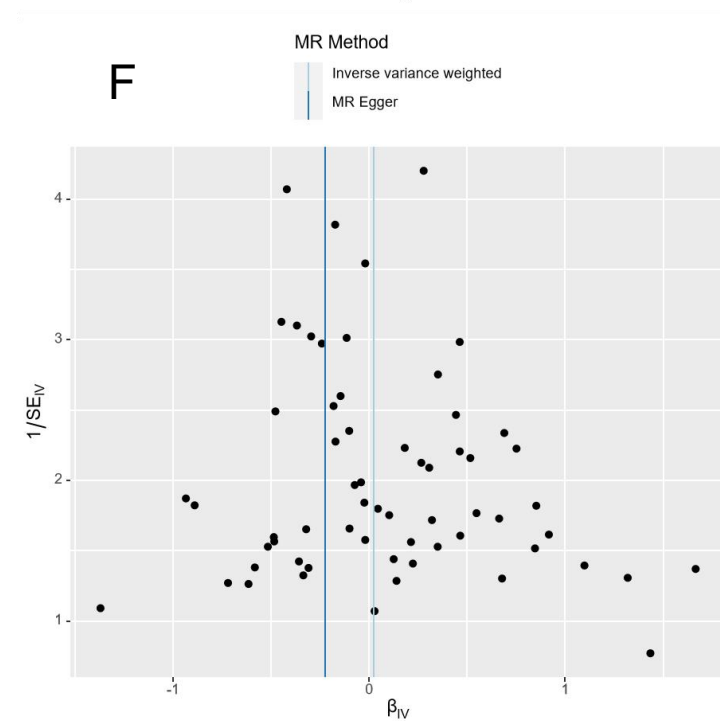

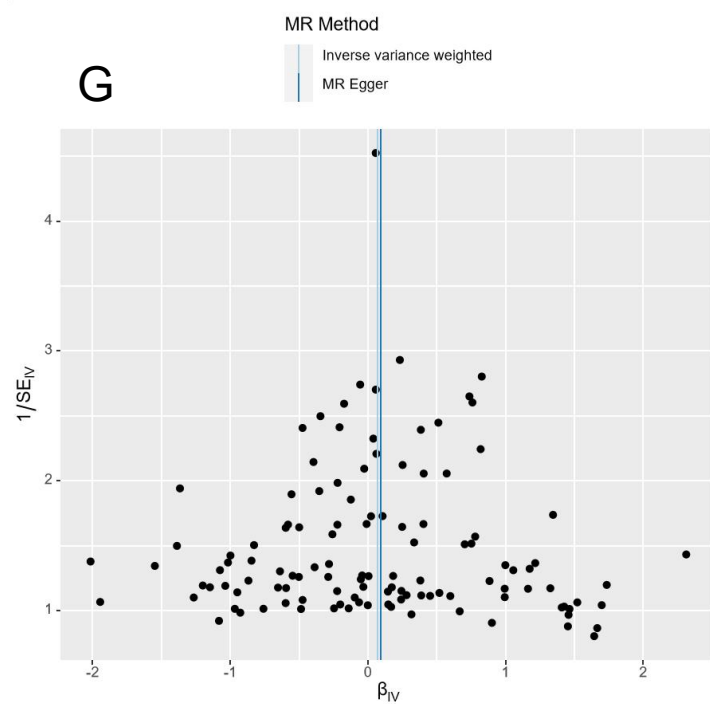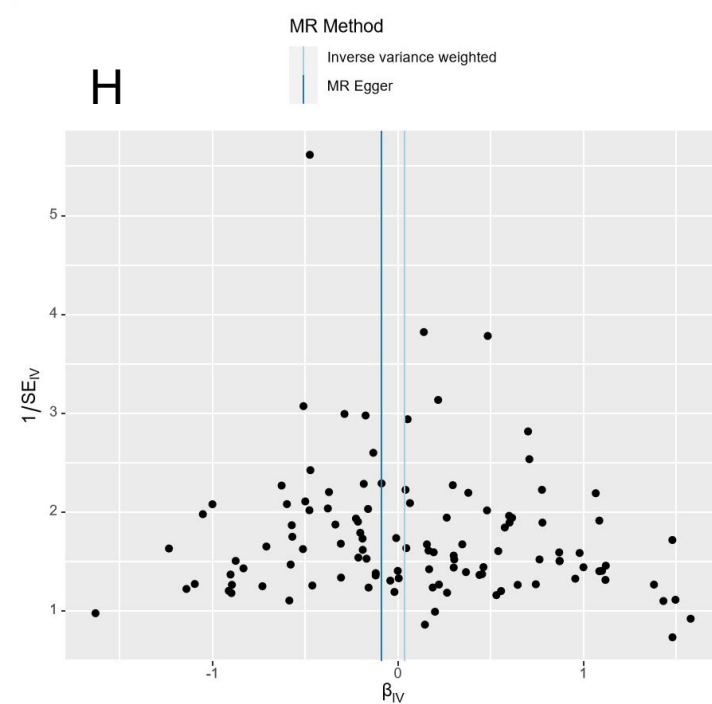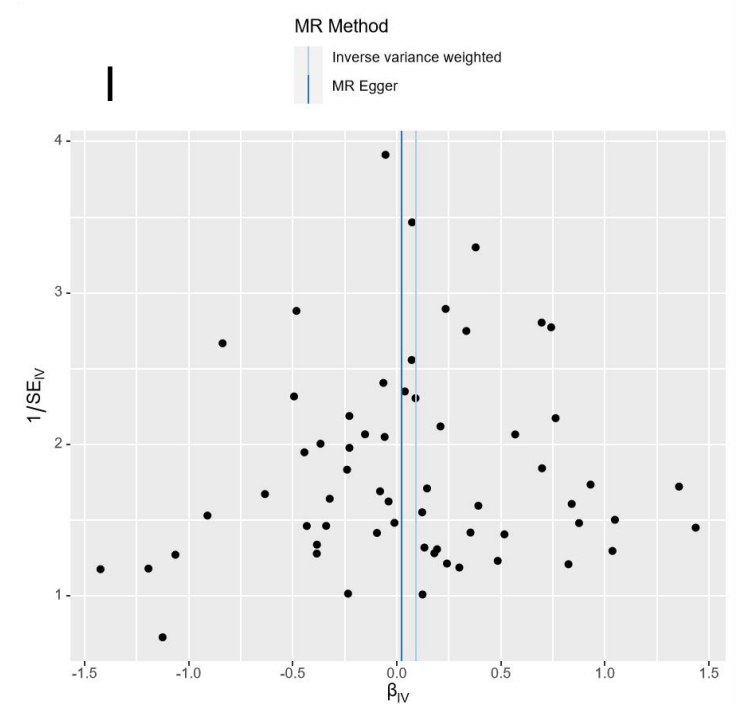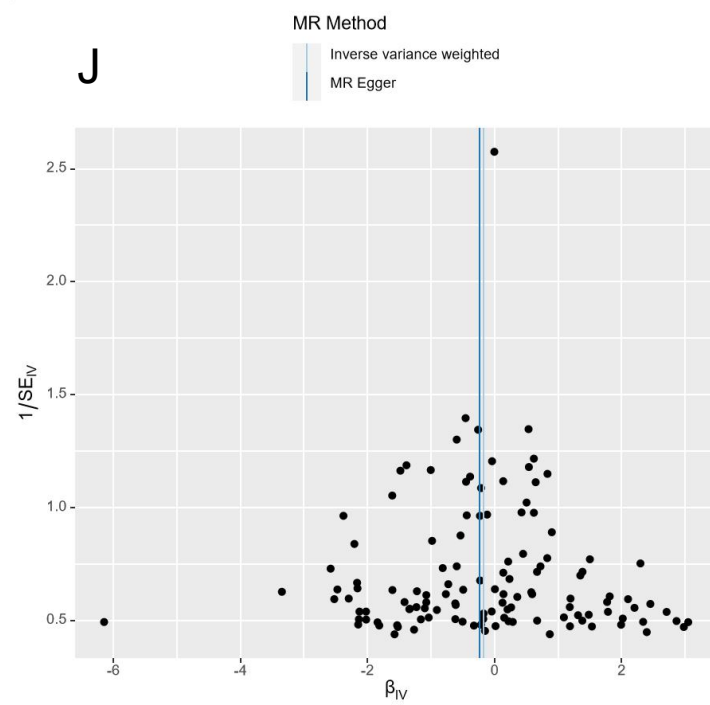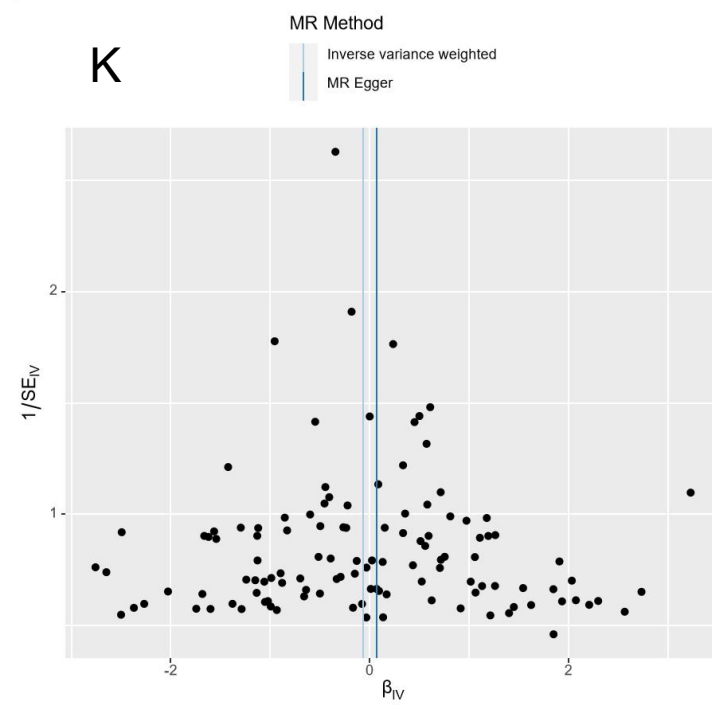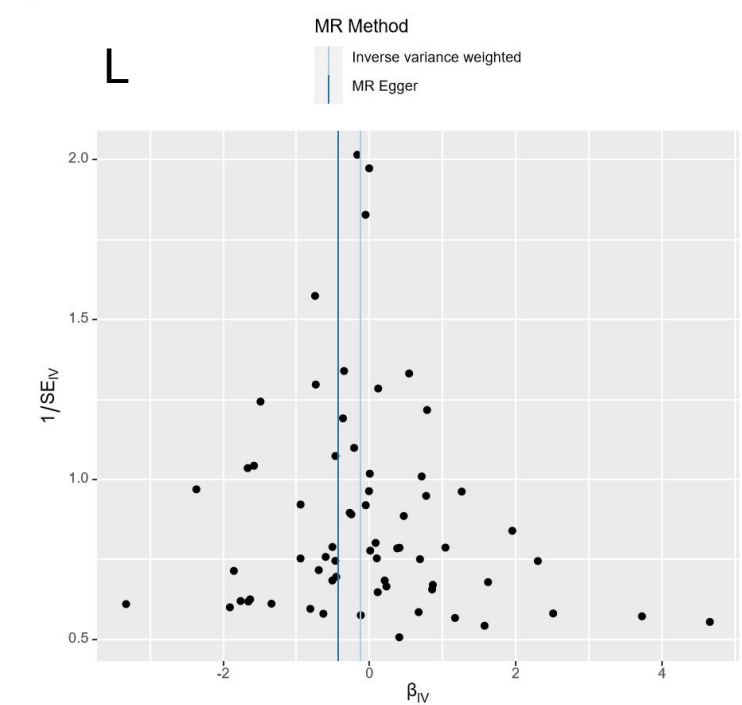

Supplement: S1 Fig — (A) IBD and scleritis, (B) CD and scleritis, (C) UC and scleritis, (D) IBD and episcleritis, (E) CD and episcleritis, (F) UC and episcleritis, (G) IBD and optic neuritis, (H) CD and optic neuritis, (I) UC and optic neuritis, (J) IBD and corneal disease, (K) CD and corneal disease, (L) UC and corneal disease. (PDF) [file pone.0316437.s001.pdf]

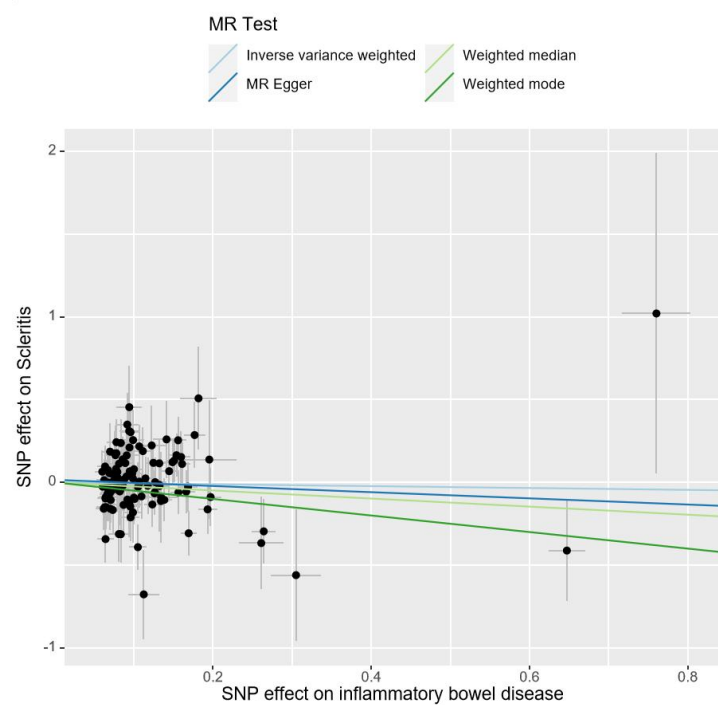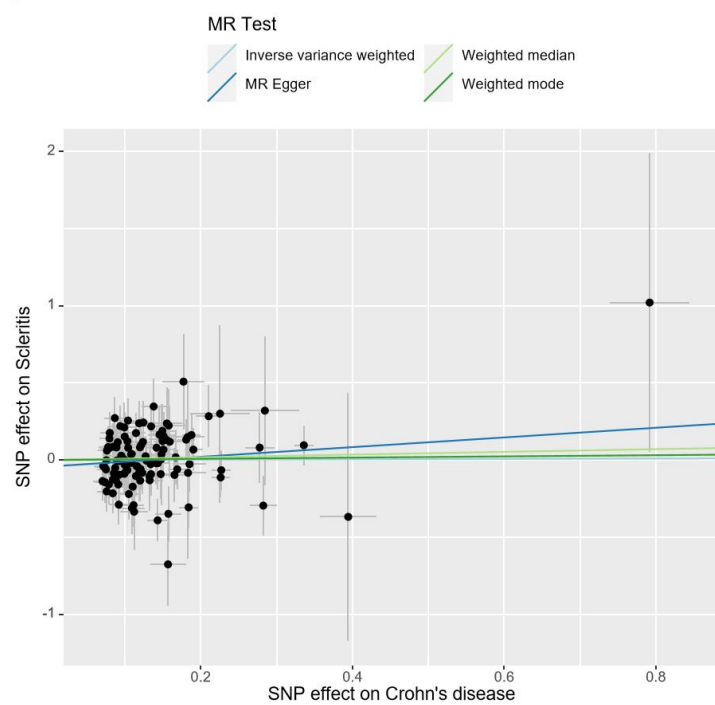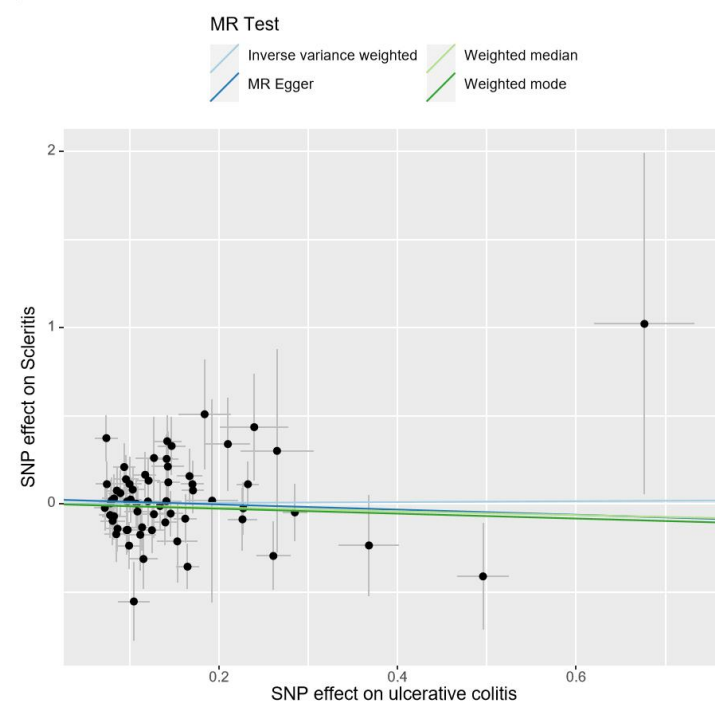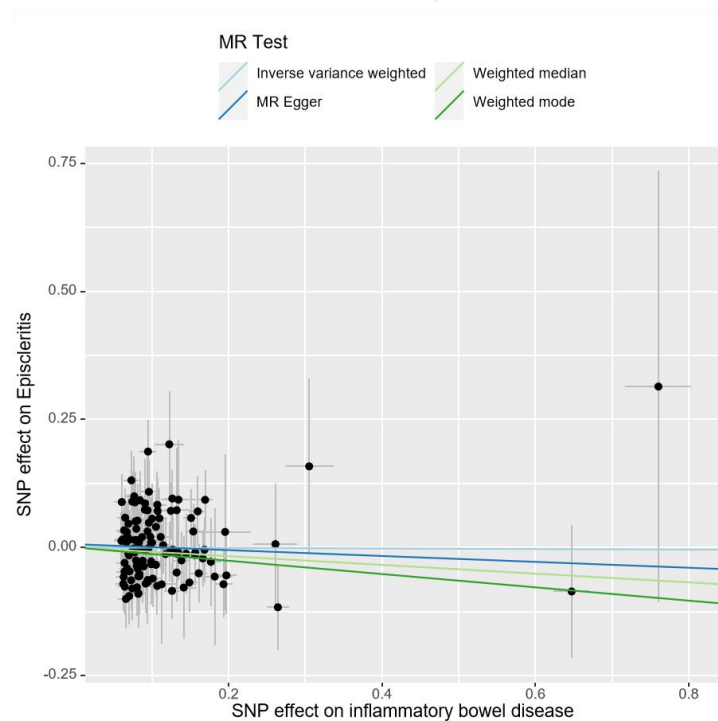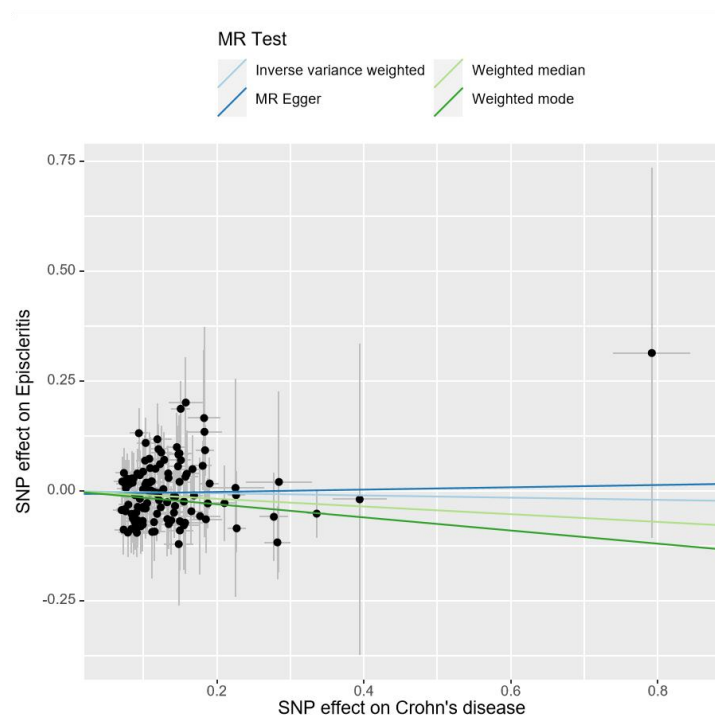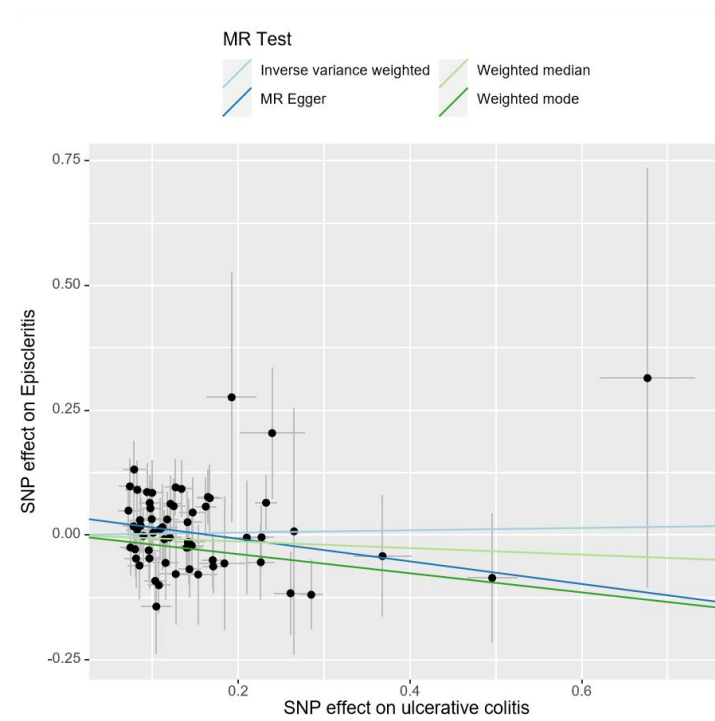

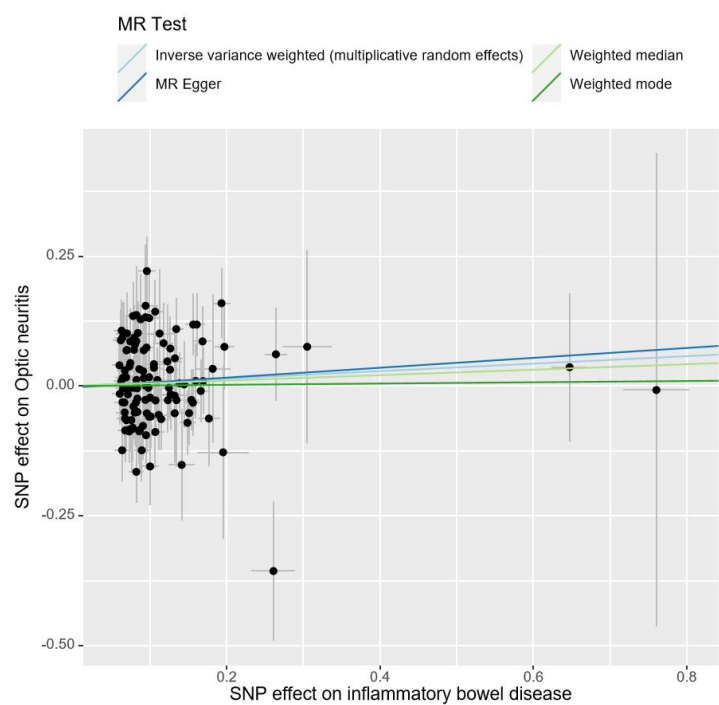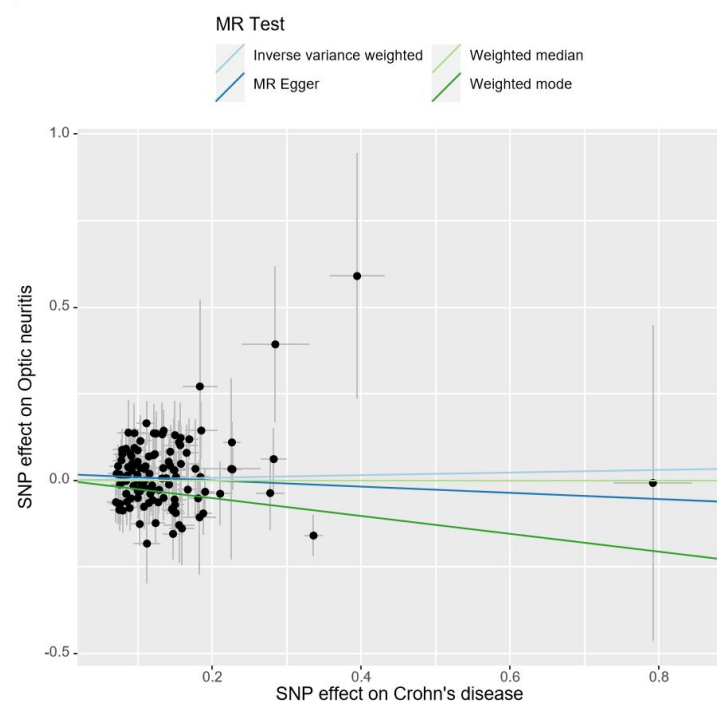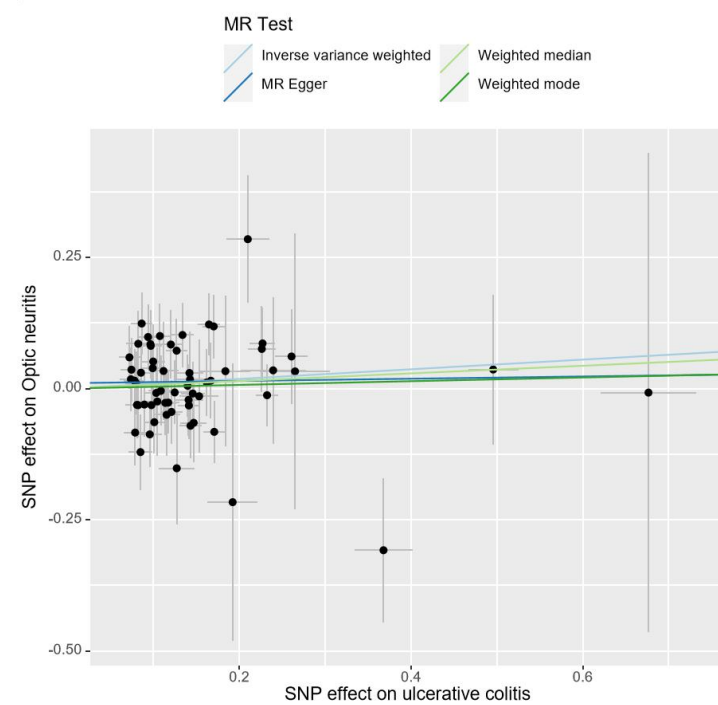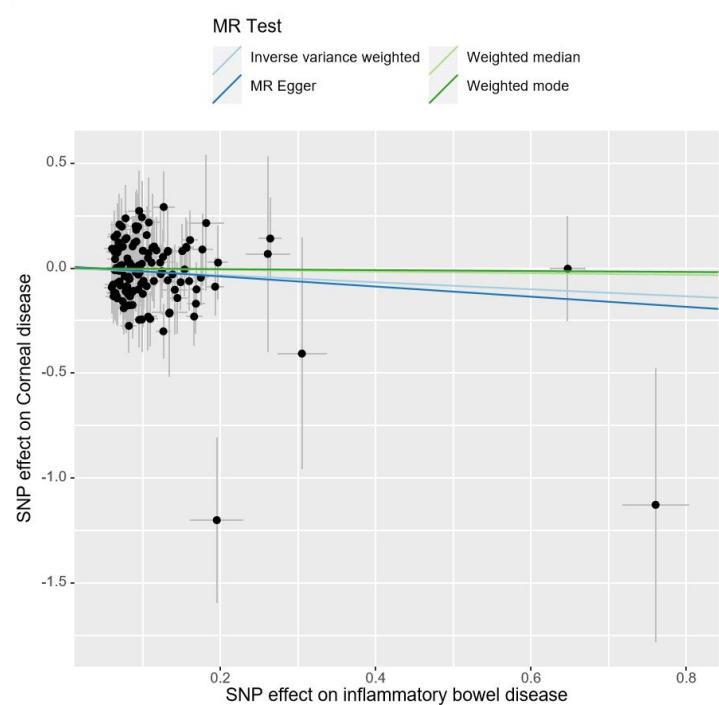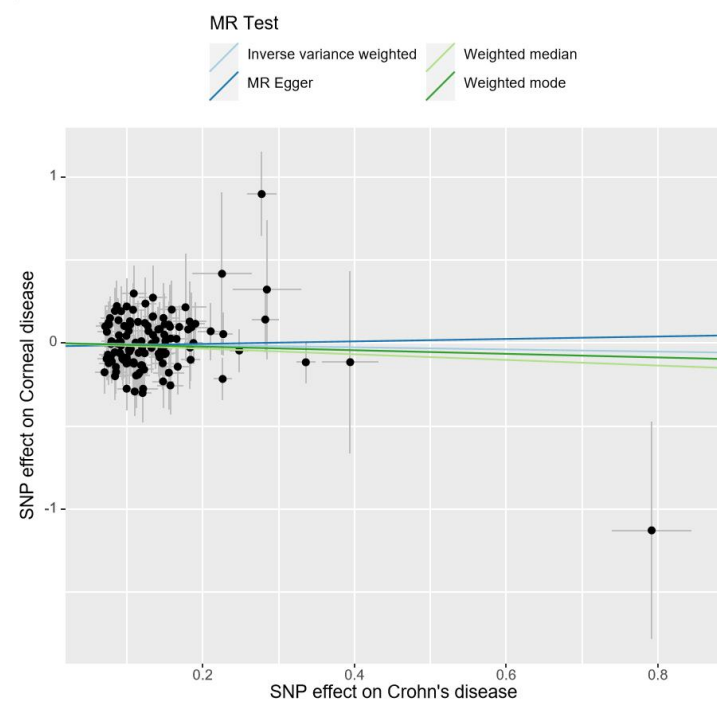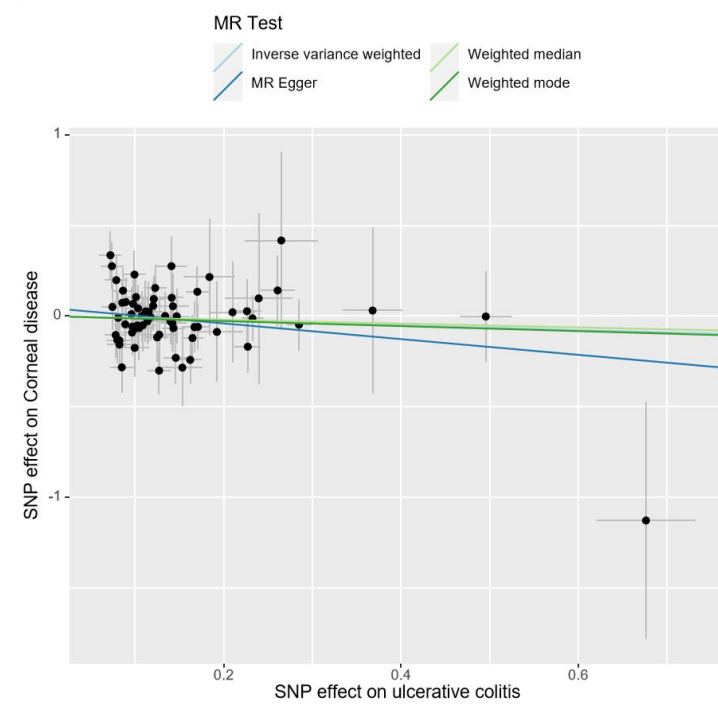

Supplement: S2 Fig — (PDF) [file pone.0316437.s002.pdf]
