# Supplementary material for: The causal effects of inflammatory bowel disease on its ocular manifestations: A Mendelian randomization study
Source: PLoS One. 2025 Mar 12;20(3):e0316437. doi: 10.1371/journal.pone.0316437 (PMC11902285; doi:10.1371/journal.pone.0316437)
Supplement: S3 Fig — (PDF) [file pone.0316437.s003.pdf]

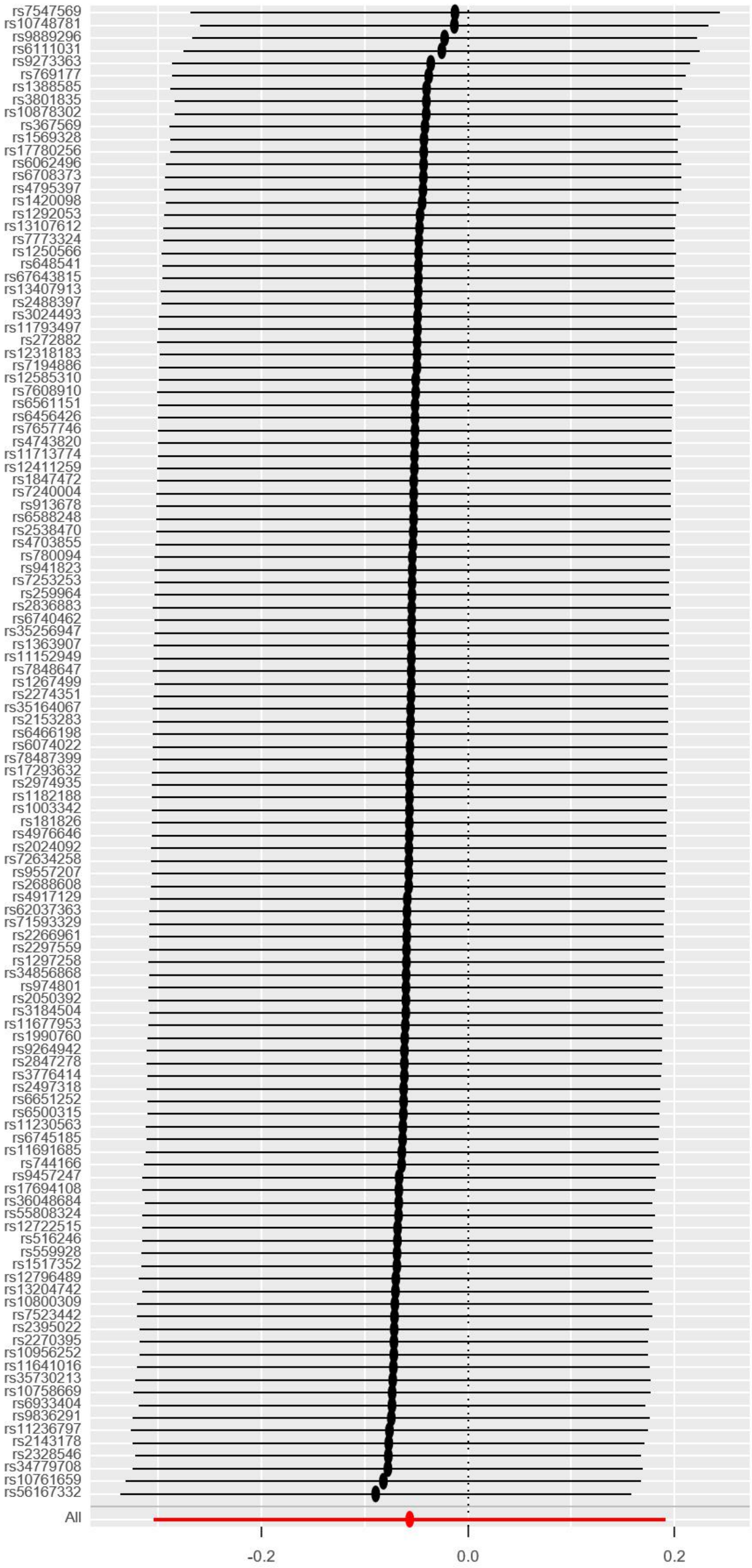

MR leave-one-out sensitivity analysis for

'inflammatory bowel disease' on 'scleritis'

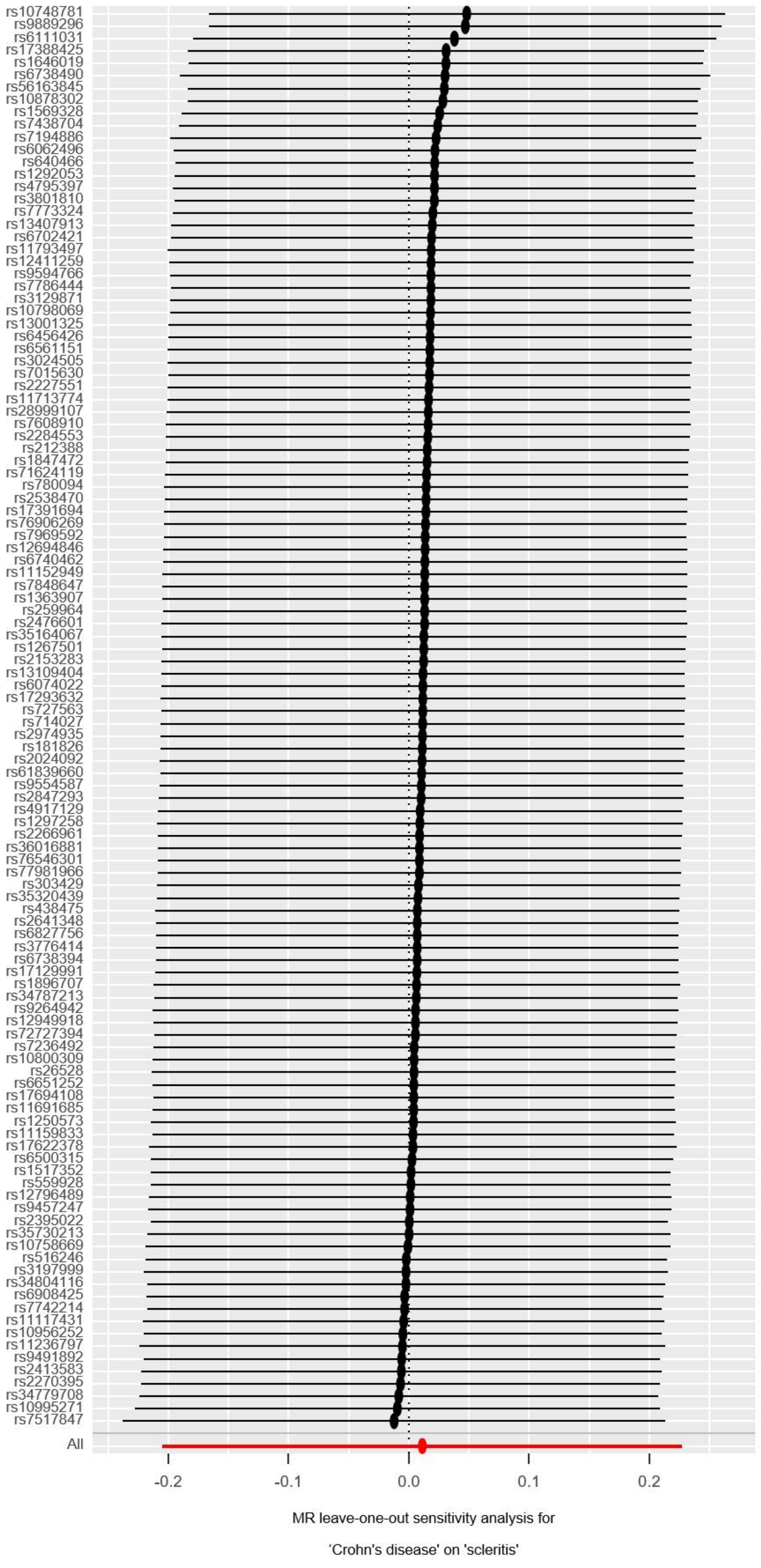
rs10748781
rs9889296
rs6111031
rs17388425
rs1646019
rs6738490
rs56163845
rs10878302
rs1569328
rs7438704
rs7194886
rs6062496
rs640466
rs1292053
rs4795397
rs3801810
rs7773324
rs13407913
rs6702421
rs11793497
rs12411259
rs9594766
rs7786444
rs3129871
rs10798069
rs13001325
rs6456426
rs6561151
rs3024505
rs7015630
rs2227551
rs11713774
rs28999107
rs7608910
rs2284553
rs212388
rs1847472
rs71624119
rs780094
rs2538470
rs17391694
rs76906269
rs7969592
rs12694846
rs6740462
rs11152949
rs7848647
rs1363907
rs259964
rs2476601
rs35164067
rs1267501
rs2153283
rs13109404
rs6074022
rs17293632
rs727563
rs714027
rs2974935
rs181826
rs2024092
rs61839660
rs9554587
rs2847293
rs4917129
rs1297258
rs2266961
rs36016881
rs76546301
rs77981966
rs303429
rs35320439
rs438475
rs2641348
rs6827756
rs3776414
rs6738394
rs17129991
rs1896707
rs34787213
rs9264942
rs12949918
rs72727394
rs7236492
rs10800309
rs26528
rs6651252
rs17694108
rs11691685
rs1250573
rs11159833
rs17622378
rs6500315
rs1517352
rs559928
rs12796489
rs9457247
rs2395022
rs35730213
rs10758669
rs516246
rs3197999
rs34804116
rs6908425
rs7742214
rs11117431
rs10956252
rs11236797
rs9491892
rs2413583
rs2270395
rs34779708
rs10995271
rs7517847
All
-0.2
-0.1
0.0
0.1
0.2
MR leave-one-out sensitivity analysis for
'Crohn's disease' on 'scleritis'

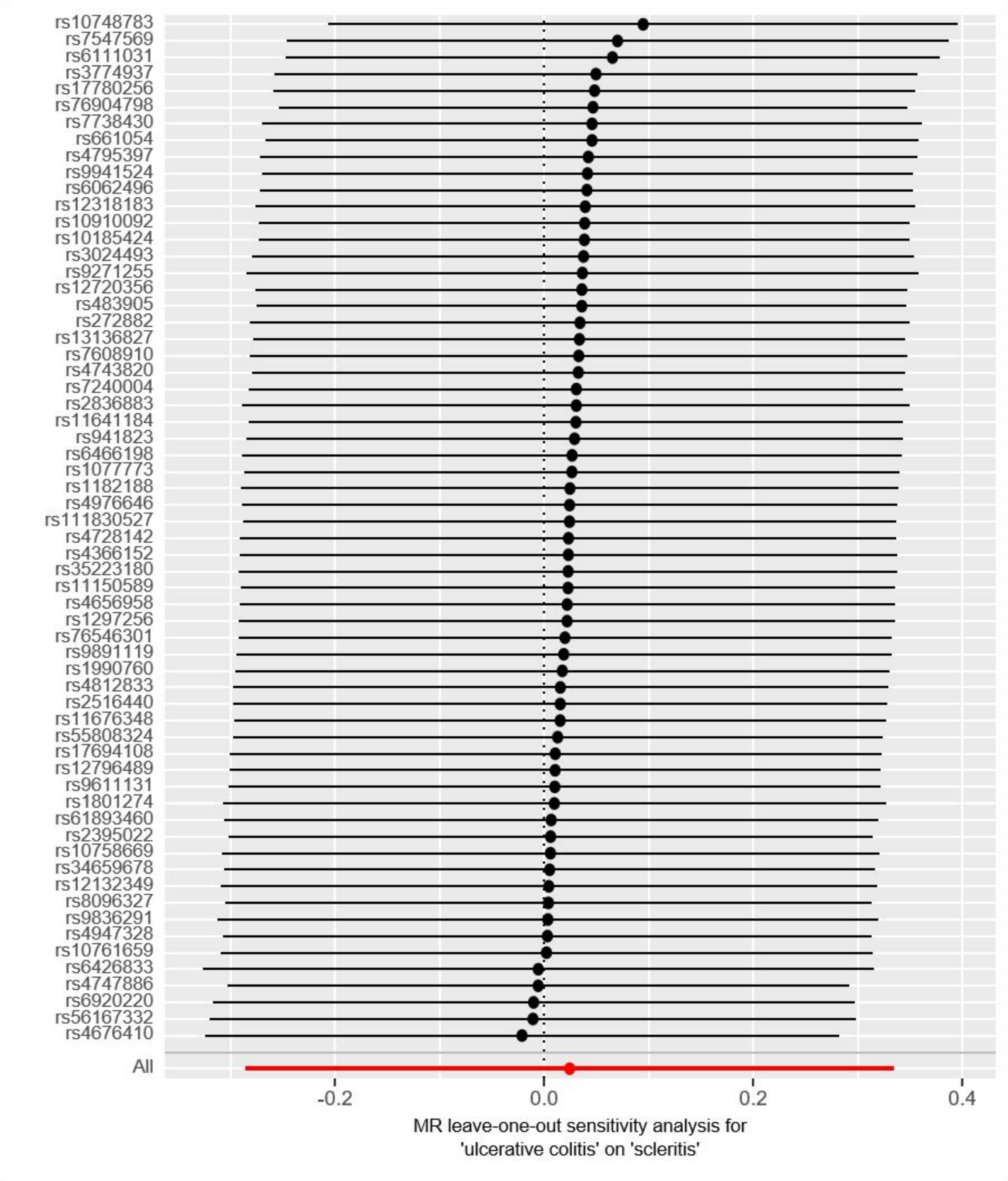
rs10748783
rs7547569
rs6111031
rs3774937
rs17780256
rs76904798
rs7738430
rs661054
rs4795397
rs9941524
rs6062496
rs12318183
rs10910092
rs10185424
rs3024493
rs9271255
rs12720356
rs483905
rs272882
rs13136827
rs7608910
rs4743820
rs7240004
rs2836883
rs11641184
rs941823
rs6466198
rs1077773
rs1182188
rs4976646
rs111830527
rs4728142
rs4366152
rs35223180
rs11150589
rs4656958
rs1297256
rs76546301
rs9891119
rs1990760
rs4812833
rs2516440
rs11676348
rs55808324
rs17694108
rs12796489
rs9611131
rs1801274
rs61893460
rs2395022
rs10758669
rs34659678
rs12132349
rs8096327
rs9836291
rs4947328
rs10761659
rs6426833
rs4747886
rs6920220
rs56167332
rs4676410
All
-0.2
0.0
0.2
0.4
MR leave-one-out sensitivity analysis for
'ulcerative colitis' on 'scleritis'

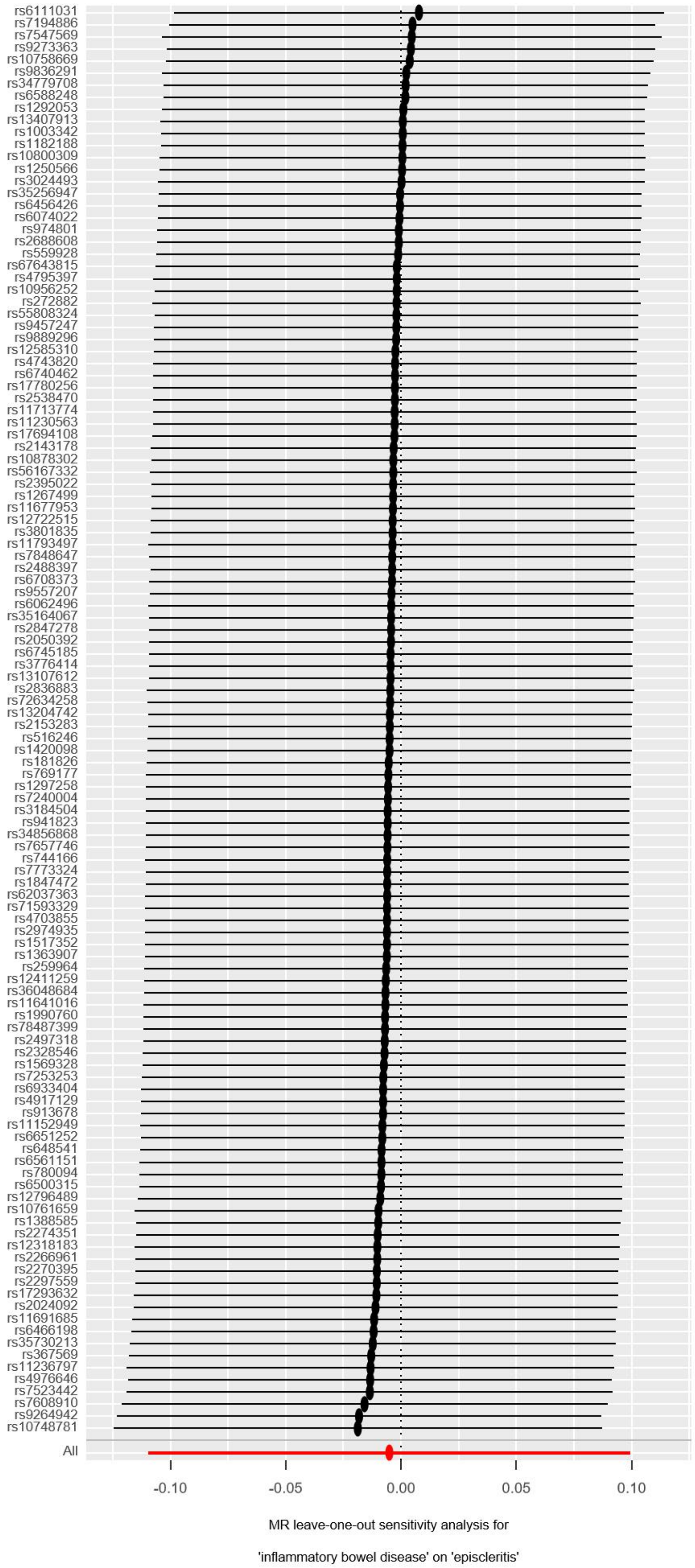
rs6111031
rs7194886
rs7547569
rs9273363
rs10758669
rs9836291
rs34779708
rs6588248
rs1292053
rs13407913
rs1003342
rs1182188
rs10800309
rs1250566
rs3024493
rs35256947
rs6456426
rs6074022
rs974801
rs2688608
rs559928
rs67643815
rs4795397
rs10956252
rs272882
rs55808324
rs9457247
rs9889296
rs12585310
rs4743820
rs6740462
rs17780256
rs2538470
rs11713774
rs11230563
rs17694108
rs2143178
rs10878302
rs56167332
rs2395022
rs1267499
rs11677953
rs12722515
rs3801835
rs11793497
rs7848647
rs2488397
rs6708373
rs9557207
rs6062496
rs35164067
rs2847278
rs2050392
rs6745185
rs3776414
rs13107612
rs2836883
rs72634258
rs13204742
rs2153283
rs516246
rs1420098
rs181826
rs769177
rs1297258
rs7240004
rs3184504
rs941823
rs34856868
rs7657746
rs744166
rs7773324
rs1847472
rs62037363
rs71593329
rs4703855
rs2974935
rs1517352
rs1363907
rs259964
rs12411259
rs36048684
rs11641016
rs1990760
rs78487399
rs2497318
rs2328546
rs1569328
rs7253253
rs6933404
rs4917129
rs913678
rs11152949
rs6651252
rs648541
rs6561151
rs780094
rs6500315
rs12796489
rs10761659
rs1388585
rs2274351
rs12318183
rs2266961
rs2270395
rs2297559
rs17293632
rs2024092
rs11691685
rs6466198
rs35730213
rs367569
rs11236797
rs4976646
rs7523442
rs7608910
rs9264942
rs10748781
All
-0.10
-0.05
0.00
0.05
0.10
MR leave-one-out sensitivity analysis for
'inflammatory bowel disease' on 'episcleritis'

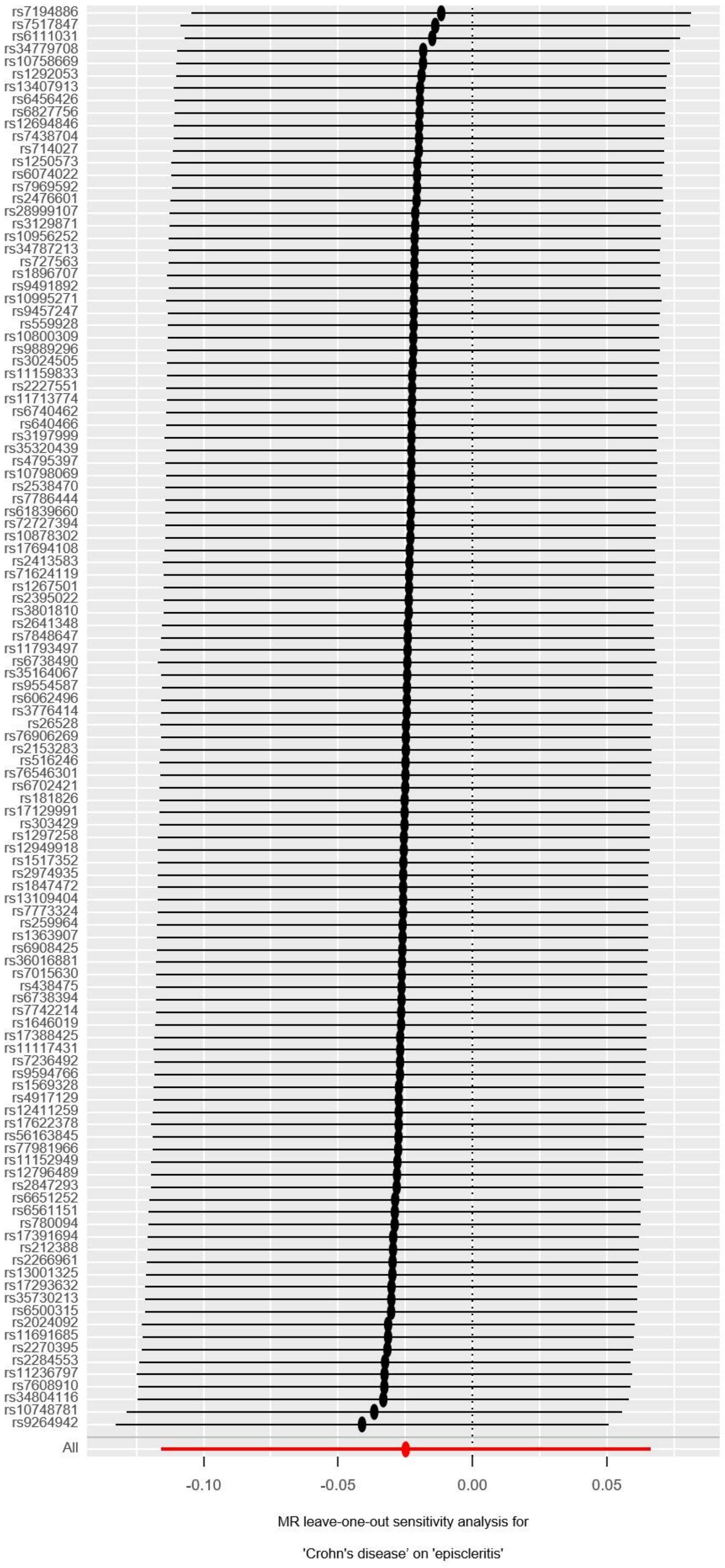
rs7194886
rs7517847
rs6111031
rs34779708
rs10758669
rs1292053
rs13407913
rs6456426
rs6827756
rs12694846
rs7438704
rs714027
rs1250573
rs6074022
rs7969592
rs2476601
rs28999107
rs3129871
rs10956252
rs34787213
rs727563
rs1896707
rs9491892
rs10995271
rs9457247
rs559928
rs10800309
rs9889296
rs3024505
rs11159833
rs2227551
rs11713774
rs6740462
rs640466
rs3197999
rs35320439
rs4795397
rs10798069
rs2538470
rs7786444
rs61839660
rs72727394
rs10878302
rs17694108
rs2413583
rs71624119
rs1267501
rs2395022
rs3801810
rs2641348
rs7848647
rs11793497
rs6738490
rs35164067
rs9554587
rs6062496
rs3776414
rs26528
rs76906269
rs2153283
rs516246
rs76546301
rs6702421
rs181826
rs17129991
rs303429
rs1297258
rs12949918
rs1517352
rs2974935
rs1847472
rs13109404
rs7773324
rs259964
rs1363907
rs6908425
rs36016881
rs7015630
rs438475
rs6738394
rs7742214
rs1646019
rs17388425
rs11117431
rs7236492
rs9594766
rs1569328
rs4917129
rs12411259
rs17622378
rs56163845
rs77981966
rs11152949
rs12796489
rs2847293
rs6651252
rs6561151
rs780094
rs17391694
rs212388
rs2266961
rs13001325
rs17293632
rs35730213
rs6500315
rs2024092
rs11691685
rs2270395
rs2284553
rs11236797
rs7608910
rs34804116
rs10748781
rs9264942
All
-0.10
-0.05
0.00
0.05
MR leave-one-out sensitivity analysis for
'Crohn's disease' on 'episcleritis'

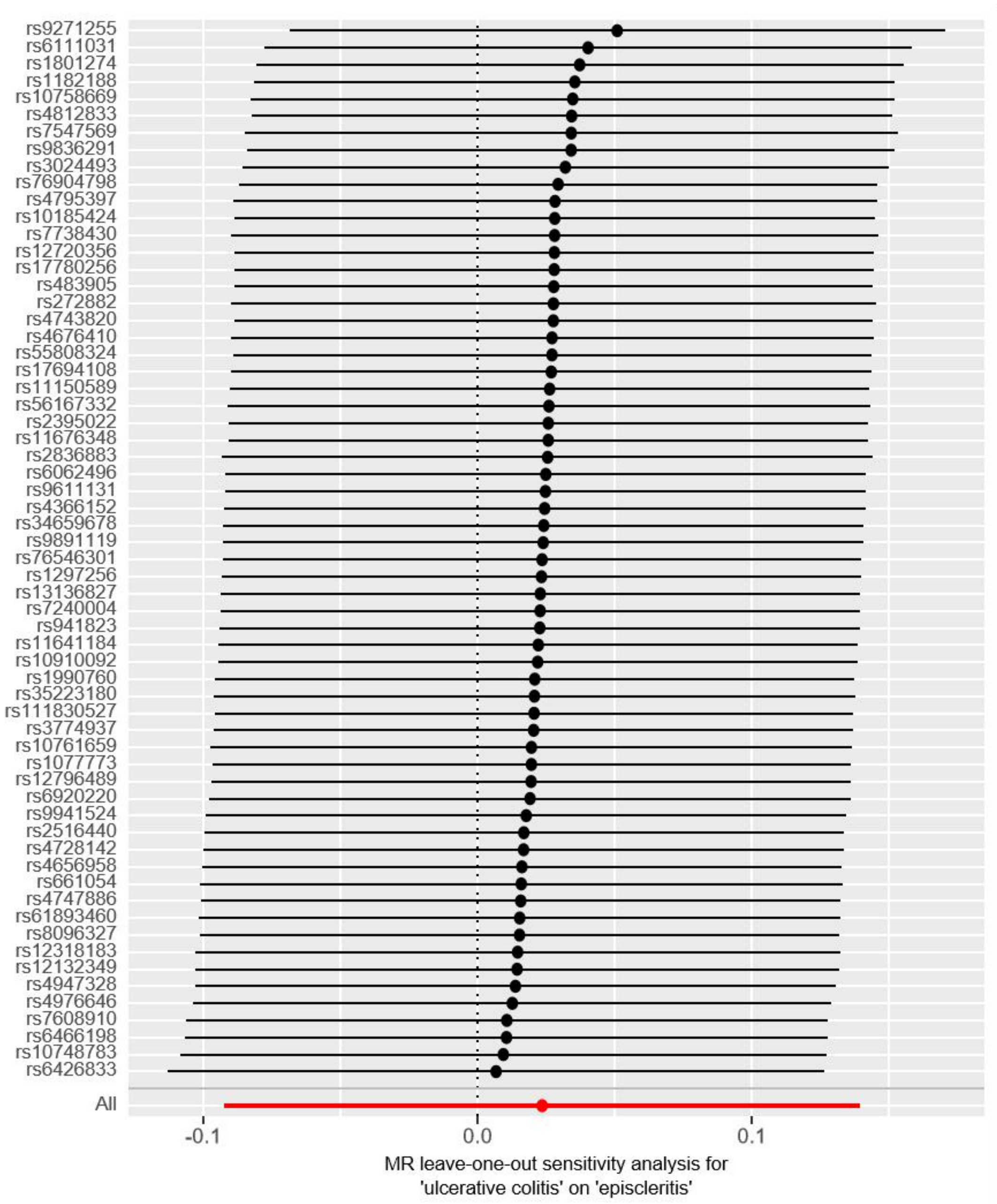
rs9271255
rs6111031
rs1801274
rs1182188
rs10758669
rs4812833
rs7547569
rs9836291
rs3024493
rs76904798
rs4795397
rs10185424
rs7738430
rs12720356
rs17780256
rs483905
rs272882
rs4743820
rs4676410
rs55808324
rs17694108
rs11150589
rs56167332
rs2395022
rs11676348
rs2836883
rs6062496
rs9611131
rs4366152
rs34659678
rs9891119
rs76546301
rs1297256
rs13136827
rs7240004
rs941823
rs11641184
rs10910092
rs1990760
rs35223180
rs111830527
rs3774937
rs10761659
rs1077773
rs12796489
rs6920220
rs9941524
rs2516440
rs4728142
rs4656958
rs661054
rs4747886
rs61893460
rs8096327
rs12318183
rs12132349
rs4947328
rs4976646
rs7608910
rs6466198
rs10748783
rs6426833
All
-0.1
0.0
0.1
MR leave-one-out sensitivity analysis for
'ulcerative colitis' on 'episcleritis'

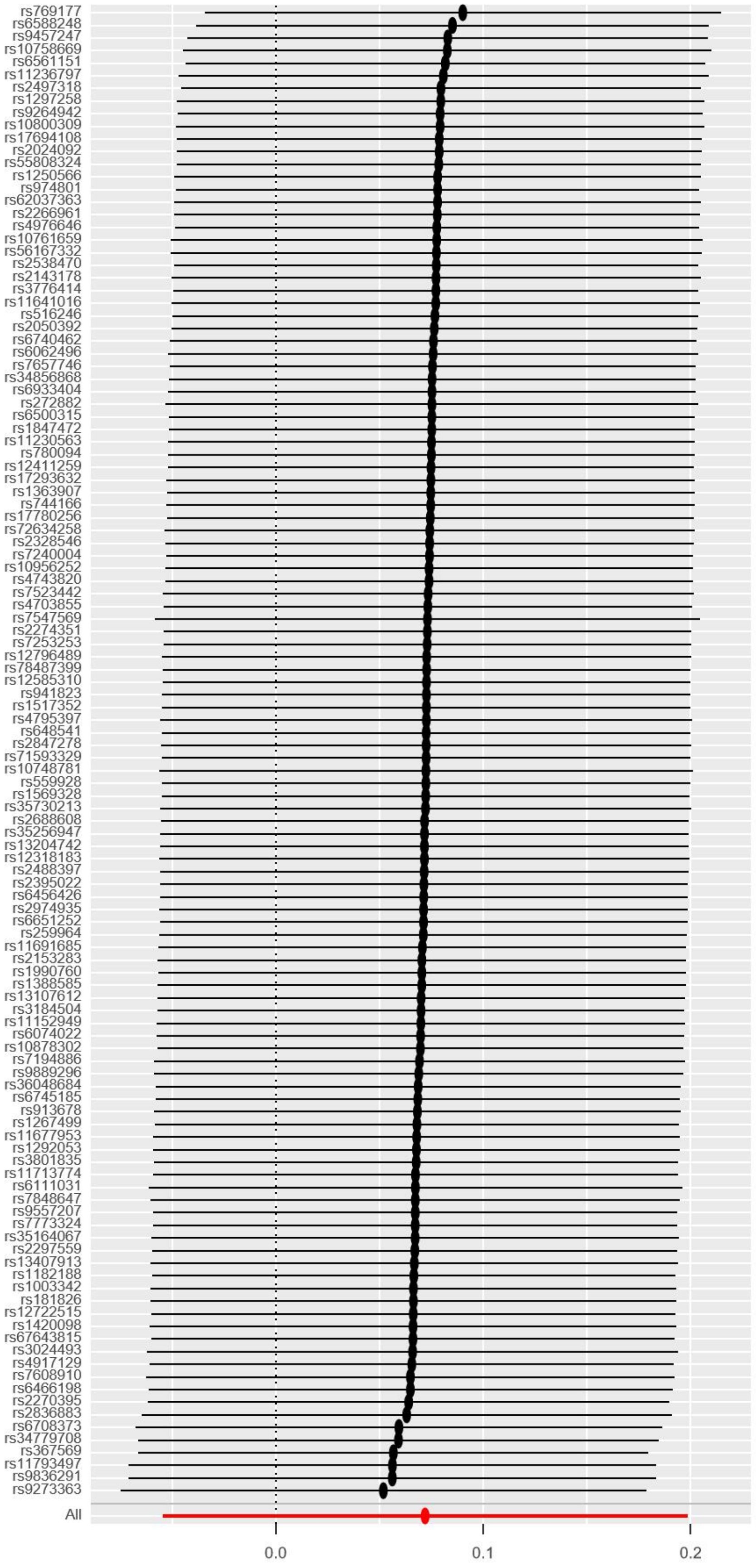

MR leave-one-out sensitivity analysis for

'inflammatory bowel disease' on 'optic neuritis'

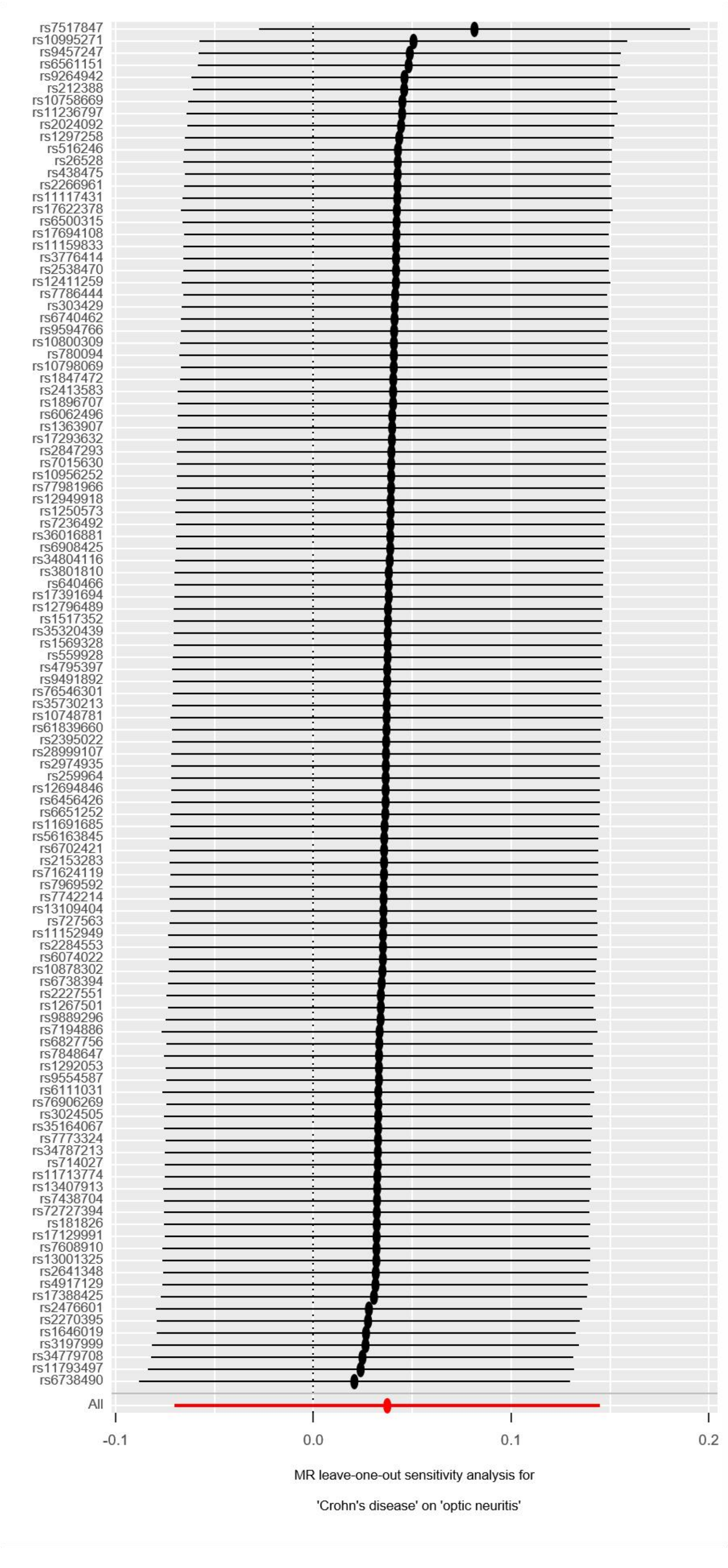
rs7517847
rs10995271
rs9457247
rs6561151
rs9264942
rs212388
rs10758669
rs11236797
rs2024092
rs1297258
rs516246
rs26528
rs438475
rs2266961
rs11117431
rs17622378
rs6500315
rs17694108
rs11159833
rs3776414
rs2538470
rs12411259
rs7786444
rs303429
rs6740462
rs9594766
rs10800309
rs780094
rs10798069
rs1847472
rs2413583
rs1896707
rs6062496
rs1363907
rs17293632
rs2847293
rs7015630
rs10956252
rs77981966
rs12949918
rs1250573
rs7236492
rs36016881
rs6908425
rs34804116
rs3801810
rs640466
rs17391694
rs12796489
rs1517352
rs35320439
rs1569328
rs559928
rs4795397
rs9491892
rs76546301
rs35730213
rs10748781
rs61839660
rs2395022
rs28999107
rs2974935
rs259964
rs12694846
rs6456426
rs6651252
rs11691685
rs56163845
rs6702421
rs2153283
rs71624119
rs7969592
rs7742214
rs13109404
rs727563
rs11152949
rs2284553
rs6074022
rs10878302
rs6738394
rs2227551
rs1267501
rs9889296
rs7194886
rs6827756
rs7848647
rs1292053
rs9554587
rs6111031
rs76906269
rs3024505
rs35164067
rs7773324
rs34787213
rs714027
rs11713774
rs13407913
rs7438704
rs72727394
rs181826
rs17129991
rs7608910
rs13001325
rs2641348
rs4917129
rs17388425
rs2476601
rs2270395
rs1646019
rs3197999
rs34779708
rs11793497
rs6738490
All
-0.1
0.0
0.1
0.2
MR leave-one-out sensitivity analysis for
'Crohn's disease' on 'optic neuritis'

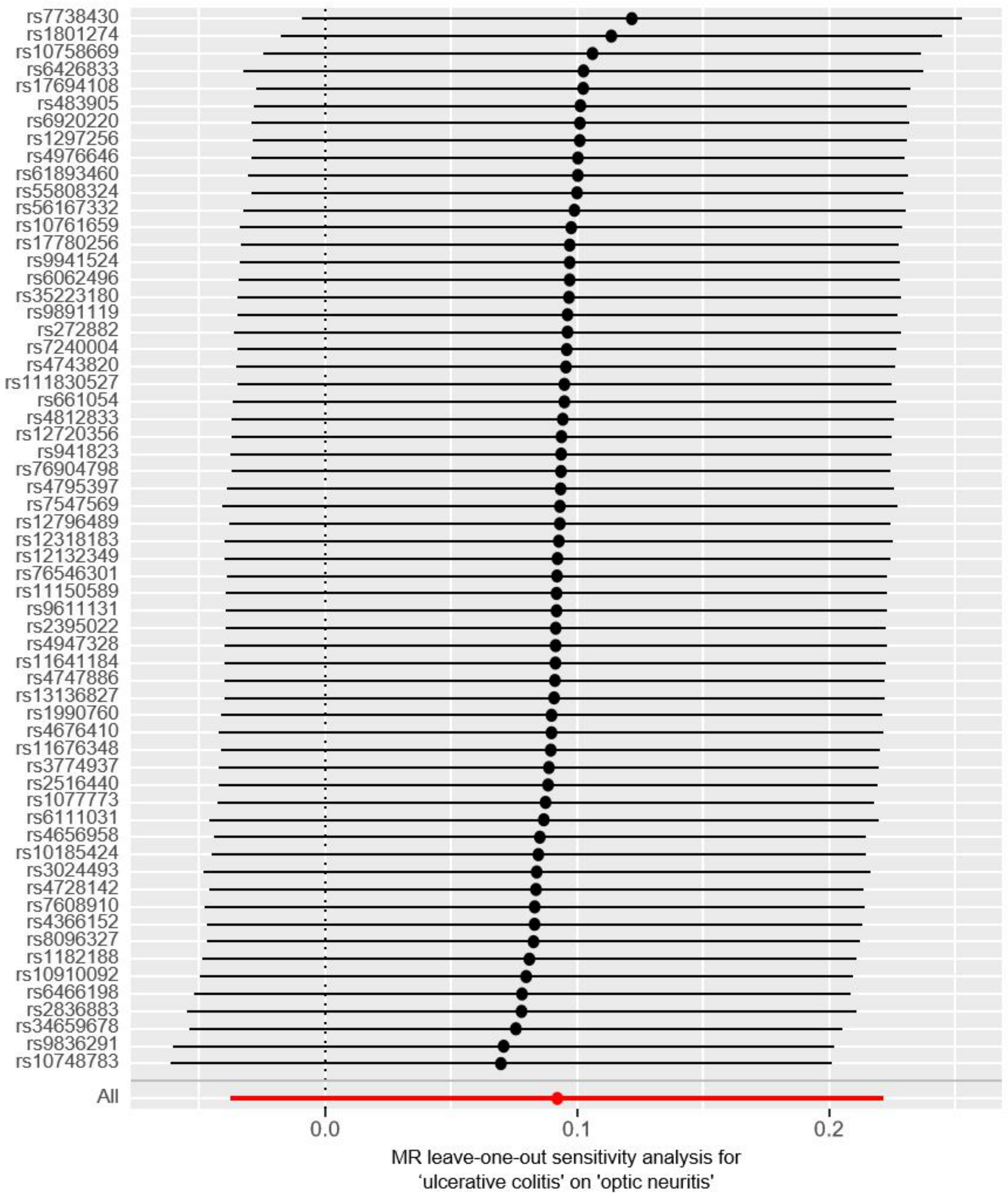
rs7738430
rs1801274
rs10758669
rs6426833
rs17694108
rs483905
rs6920220
rs1297256
rs4976646
rs61893460
rs55808324
rs56167332
rs10761659
rs17780256
rs9941524
rs6062496
rs35223180
rs9891119
rs272882
rs7240004
rs4743820
rs111830527
rs661054
rs4812833
rs12720356
rs941823
rs76904798
rs4795397
rs7547569
rs12796489
rs12318183
rs12132349
rs76546301
rs11150589
rs9611131
rs2395022
rs4947328
rs11641184
rs4747886
rs13136827
rs1990760
rs4676410
rs11676348
rs3774937
rs2516440
rs1077773
rs6111031
rs4656958
rs10185424
rs3024493
rs4728142
rs7608910
rs4366152
rs8096327
rs1182188
rs10910092
rs6466198
rs2836883
rs34659678
rs9836291
rs10748783
All
0.0
0.1
0.2
MR leave-one-out sensitivity analysis for
'ulcerative colitis' on 'optic neuritis'

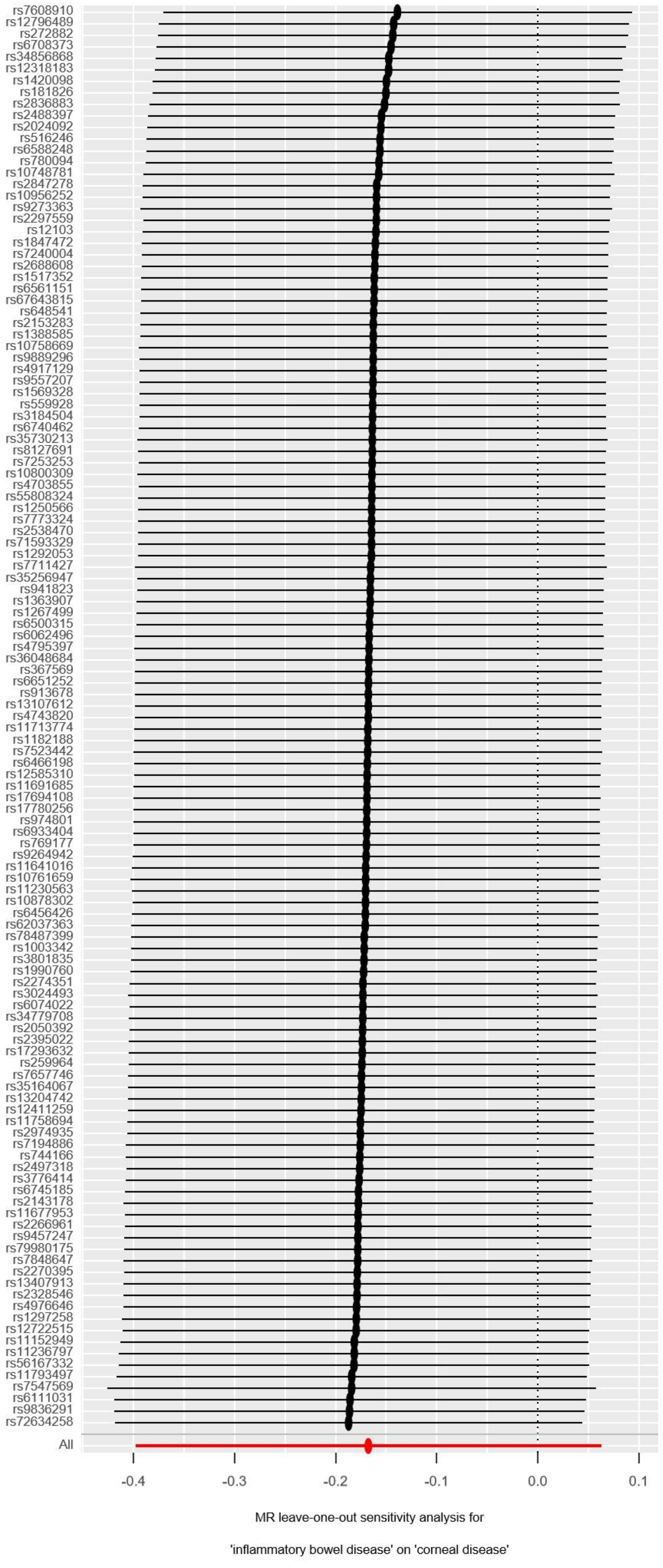
rs7608910
rs12796489
rs272882
rs6708373
rs34856868
rs12318183
rs1420098
rs181826
rs2836883
rs2488397
rs2024092
rs516246
rs6588248
rs780094
rs10748781
rs2847278
rs10956252
rs9273363
rs2297559
rs12103
rs1847472
rs7240004
rs2688608
rs1517352
rs6561151
rs67643815
rs648541
rs2153283
rs1388585
rs10758669
rs9889296
rs4917129
rs9557207
rs1569328
rs559928
rs3184504
rs6740462
rs35730213
rs8127691
rs7253253
rs10800309
rs4703855
rs55808324
rs1250566
rs7773324
rs2538470
rs71593329
rs1292053
rs7711427
rs35256947
rs941823
rs1363907
rs1267499
rs6500315
rs6062496
rs4795397
rs36048684
rs367569
rs6651252
rs913678
rs13107612
rs4743820
rs11713774
rs1182188
rs7523442
rs6466198
rs12585310
rs11691685
rs17694108
rs17780256
rs974801
rs6933404
rs769177
rs9264942
rs11641016
rs10761659
rs11230563
rs10878302
rs6456426
rs62037363
rs78487399
rs1003342
rs3801835
rs1990760
rs2274351
rs3024493
rs6074022
rs34779708
rs2050392
rs2395022
rs17293632
rs259964
rs7657746
rs35164067
rs13204742
rs12411259
rs11758694
rs2974935
rs7194886
rs744166
rs2497318
rs3776414
rs6745185
rs2143178
rs11677953
rs2266961
rs9457247
rs79980175
rs7848647
rs2270395
rs13407913
rs2328546
rs4976646
rs1297258
rs12722515
rs11152949
rs11236797
rs56167332
rs11793497
rs7547569
rs6111031
rs9836291
rs72634258
All
-0.4
-0.3
-0.2
-0.1
0.0
0.1
MR leave-one-out sensitivity analysis for
'inflammatory bowel disease' on 'corneal disease'

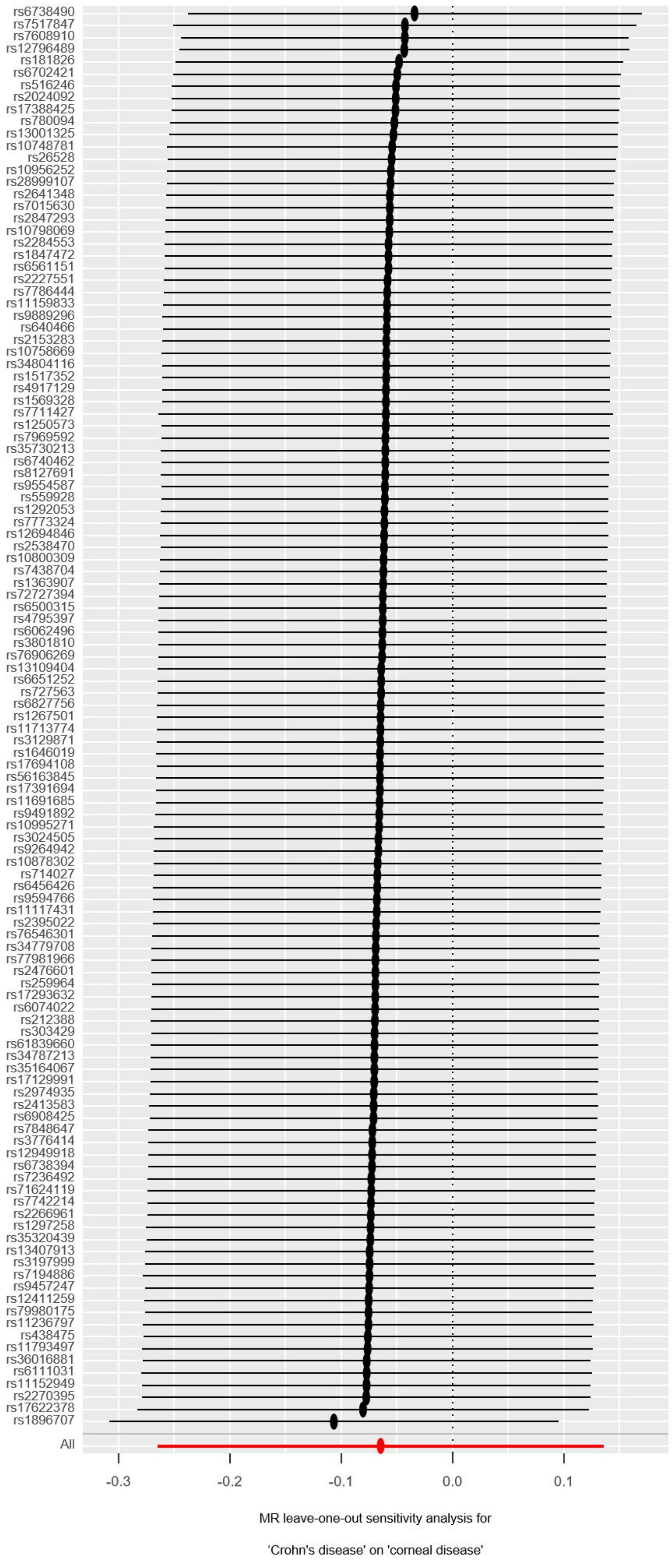

rs6738490
rs7517847
rs7608910
rs12796489
rs181826
rs6702421
rs516246
rs2024092
rs17388425
rs780094
rs13001325
rs10748781
rs26528
rs10956252
rs28999107
rs2641348
rs7015630
rs2847293
rs10798069
rs2284553
rs1847472
rs6561151
rs2227551
rs7786444
rs11159833
rs9889296
rs640466
rs2153283
rs10758669
rs34804116
rs1517352
rs4917129
rs1569328
rs7711427
rs1250573
rs7969592
rs35730213
rs6740462
rs8127691
rs9554587
rs559928
rs1292053
rs7773324
rs12694846
rs2538470
rs10800309
rs7438704
rs1363907
rs72727394
rs6500315
rs4795397
rs6062496
rs3801810
rs76906269
rs13109404
rs6651252
rs727563
rs6827756
rs1267501
rs11713774
rs3129871
rs1646019
rs17694108
rs56163845
rs17391694
rs11691685
rs9491892
rs10995271
rs3024505
rs9264942
rs10878302
rs714027
rs6456426
rs9594766
rs11117431
rs2395022
rs76546301
rs34779708
rs77981966
rs2476601
rs259964
rs17293632
rs6074022
rs212388
rs303429
rs61839660
rs34787213
rs35164067
rs17129991
rs2974935
rs2413583
rs6908425
rs7848647
rs3776414
rs12949918
rs6738394
rs7236492
rs71624119
rs7742214
rs2266961
rs1297258
rs35320439
rs13407913
rs3197999
rs7194886
rs9457247
rs12411259
rs79980175
rs11236797
rs438475
rs11793497
rs36016881
rs6111031
rs11152949
rs2270395
rs17622378
rs1896707
All
-0.3
-0.2
-0.1
0.0
0.1
MR leave-one-out sensitivity analysis for
'Crohn's disease' on 'corneal disease'

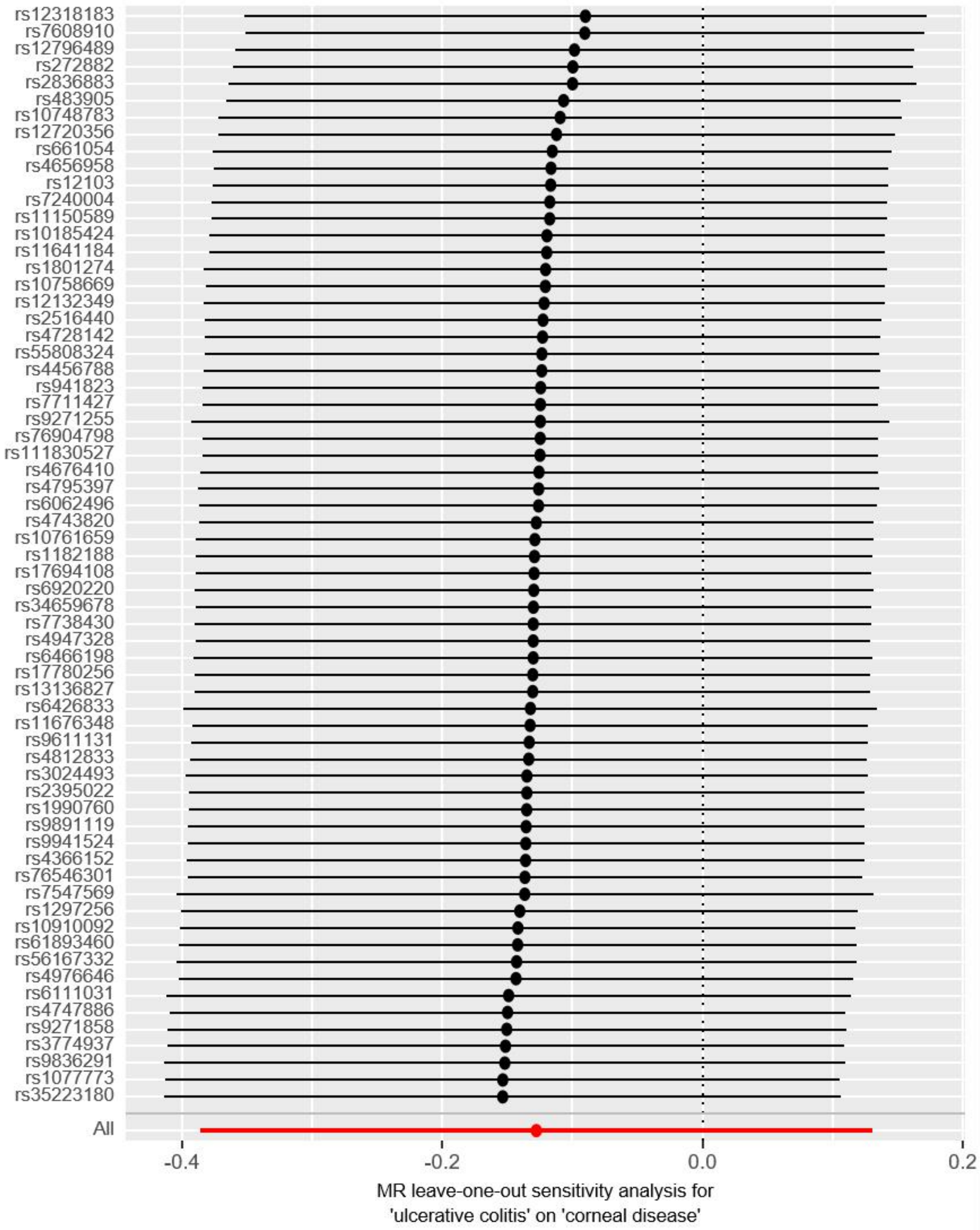

rs12318183
rs7608910
rs12796489
rs272882
rs2836883
rs483905
rs10748783
rs12720356
rs661054
rs4656958
rs12103
rs7240004
rs11150589
rs10185424
rs11641184
rs1801274
rs10758669
rs12132349
rs2516440
rs4728142
rs55808324
rs4456788
rs941823
rs7711427
rs9271255
rs76904798
rs111830527
rs4676410
rs4795397
rs6062496
rs4743820
rs10761659
rs1182188
rs17694108
rs6920220
rs34659678
rs7738430
rs4947328
rs6466198
rs17780256
rs13136827
rs6426833
rs11676348
rs9611131
rs4812833
rs3024493
rs2395022
rs1990760
rs9891119
rs9941524
rs4366152
rs76546301
rs7547569
rs1297256
rs10910092
rs61893460
rs56167332
rs4976646
rs6111031
rs4747886
rs9271858
rs3774937
rs9836291
rs1077773
rs35223180
All
-0.4
-0.2
0.0
0.2
MR leave-one-out sensitivity analysis for
'ulcerative colitis' on 'corneal disease'
