# Supplementary material for: The causal effects of inflammatory bowel disease on its ocular manifestations: A Mendelian randomization study
Source: PLoS One. 2025 Mar 12;20(3):e0316437. doi: 10.1371/journal.pone.0316437 (PMC11902285; doi:10.1371/journal.pone.0316437)
Supplement: S8 Table — (DOC) [file pone.0316437.s011.doc]

**S8 Table. The general information of the chosen genetic IVs for CD on scleritis.**

| SNP | effect_allele.  exposure | other_allele.  exposure | beta.  exposure | eaf.  exposure | se.  exposure | samplesize.  exposure | pval.  exposure | R2 | F |
| --- | --- | --- | --- | --- | --- | --- | --- | --- | --- |
| rs10748781 | A | C | -0.183963 | 0.5733 | 0.0121813 | 51874 | 1.57E-51 | 0.01655753 | 873.3324163 |
| rs10758669 | A | C | -0.14975 | 0.6504 | 0.012299 | 51874 | 4.19E-34 | 0.010198014 | 534.4416384 |
| rs10798069 | T | G | -0.070387 | 0.4925 | 0.0119822 | 51874 | 4.25E-09 | 0.002476608 | 128.7855366 |
| rs10800309 | G | A | -0.0904086 | 0.6578 | 0.0126389 | 51874 | 8.48E-13 | 0.003679793 | 191.5831955 |
| rs10878302 | A | T | 0.15677 | 0.92855 | 0.023765 | 51874 | 4.20E-11 | 0.003261095 | 169.7129657 |
| rs10956252 | G | C | 0.118641 | 0.6189 | 0.0123641 | 51874 | 8.34E-22 | 0.006639862 | 346.7251054 |
| rs10995271 | C | G | -0.187653 | 0.6098 | 0.012242 | 51874 | 4.92E-53 | 0.01675775 | 884.0730782 |
| rs11117431 | G | A | -0.14927 | 0.1978 | 0.0164404 | 51874 | 1.09E-19 | 0.007071052 | 369.4016465 |
| rs11152949 | G | A | 0.133849 | 0.3195 | 0.0128549 | 51874 | 2.18E-25 | 0.007790391 | 407.2759956 |
| rs11159833 | T | C | 0.155227 | 0.08681 | 0.0207601 | 51874 | 7.59E-14 | 0.003820282 | 198.9256222 |
| rs11236797 | A | C | 0.180737 | 0.4444 | 0.0120572 | 51874 | 8.54E-51 | 0.016130968 | 850.4643698 |
| rs11691685 | G | A | -0.157687 | 0.08024 | 0.0233162 | 51874 | 1.35E-11 | 0.003670179 | 191.0808125 |
| rs11713774 | C | T | 0.132662 | 0.1427 | 0.0171654 | 51874 | 1.09E-14 | 0.004306058 | 224.3298168 |
| rs11793497 | G | A | 0.168922 | 0.423 | 0.0121801 | 51874 | 9.80E-44 | 0.013928957 | 732.729022 |
| rs12411259 | A | G | 0.134364 | 0.2401 | 0.0137443 | 51874 | 1.43E-22 | 0.006587861 | 343.9917124 |
| rs1250573 | A | G | -0.141693 | 0.3155 | 0.0134477 | 51874 | 5.86E-26 | 0.008671607 | 453.7483417 |
| rs1267501 | C | T | 0.0871787 | 0.8106 | 0.0151984 | 51874 | 9.69E-09 | 0.002333659 | 121.3346997 |
| rs12694846 | G | A | 0.115366 | 0.2593 | 0.0136242 | 51874 | 2.50E-17 | 0.005112467 | 266.556656 |
| rs12796489 | A | C | -0.79177 | 0.02286 | 0.0526931 | 51874 | 4.96E-51 | 0.028006646 | 1494.620023 |
| rs1292053 | G | A | 0.0911854 | 0.442 | 0.0118917 | 51874 | 1.75E-14 | 0.004101447 | 213.626424 |
| rs12949918 | C | T | -0.104177 | 0.4185 | 0.0123587 | 51874 | 3.47E-17 | 0.005282249 | 275.4558472 |
| rs1297258 | T | C | -0.127246 | 0.4249 | 0.0122169 | 51874 | 2.11E-25 | 0.007913131 | 413.7439577 |
| rs13001325 | T | C | -0.122687 | 0.3757 | 0.0125708 | 51874 | 1.68E-22 | 0.007060925 | 368.8688751 |
| rs13109404 | G | T | 0.183131 | 0.06362 | 0.0236023 | 51874 | 8.56E-15 | 0.003995761 | 208.0996404 |
| rs13407913 | G | A | 0.114508 | 0.4306 | 0.0119519 | 51874 | 9.64E-22 | 0.006429736 | 335.6816108 |
| rs1363907 | A | G | 0.102575 | 0.4212 | 0.0125984 | 51874 | 3.89E-16 | 0.005130148 | 267.4832891 |
| rs1517352 | C | A | 0.0800097 | 0.6048 | 0.0124517 | 51874 | 1.31E-10 | 0.003060159 | 159.22382 |
| rs1569328 | T | C | -0.109215 | 0.1702 | 0.0167191 | 51874 | 6.47E-11 | 0.003369206 | 175.3582704 |
| rs1646019 | T | C | -0.111379 | 0.303 | 0.013383 | 51874 | 8.62E-17 | 0.005239768 | 273.2288845 |
| rs17129991 | T | C | -0.284461 | 0.02246 | 0.0450901 | 51874 | 2.81E-10 | 0.003553201 | 184.9688628 |
| rs17293632 | T | C | 0.128384 | 0.2364 | 0.0139602 | 51874 | 3.70E-20 | 0.005950661 | 310.5204774 |
| rs17388425 | G | C | -0.157253 | 0.1598 | 0.0172142 | 51874 | 6.54E-20 | 0.006640294 | 346.7478528 |
| rs17391694 | T | C | -0.119051 | 0.1226 | 0.0199954 | 51874 | 2.62E-09 | 0.003049188 | 158.6512335 |
| rs17622378 | G | A | 0.190197 | 0.4186 | 0.0120781 | 51874 | 7.17E-56 | 0.017608063 | 929.7362745 |
| rs17694108 | A | G | 0.0795895 | 0.2797 | 0.0134523 | 51874 | 3.29E-09 | 0.002552392 | 132.7364901 |
| rs181826 | A | C | 0.0995948 | 0.6266 | 0.0127047 | 51874 | 4.53E-15 | 0.004641603 | 241.8920172 |
| rs1847472 | A | C | -0.0853854 | 0.3421 | 0.0132299 | 51874 | 1.09E-10 | 0.003281785 | 170.793284 |
| rs1896707 | T | C | 0.277596 | 0.08264 | 0.0196734 | 51874 | 3.29E-45 | 0.011683864 | 613.2303189 |
| rs2024092 | A | G | 0.147664 | 0.2162 | 0.0143378 | 51874 | 7.13E-25 | 0.007389928 | 386.184214 |
| rs212388 | T | C | -0.102377 | 0.6043 | 0.0124319 | 51874 | 1.80E-16 | 0.005012489 | 261.3176849 |
| rs2153283 | A | C | -0.108804 | 0.217 | 0.0155227 | 51874 | 2.39E-12 | 0.004022918 | 209.5196998 |
| rs2227551 | T | G | 0.0994456 | 0.7288 | 0.0137489 | 51874 | 4.72E-13 | 0.003909302 | 203.5791507 |
| rs2266961 | G | C | -0.108108 | 0.8035 | 0.0149511 | 51874 | 4.80E-13 | 0.003690576 | 192.1466655 |
| rs2270395 | T | C | 0.124328 | 0.7616 | 0.0144786 | 51874 | 8.93E-18 | 0.005613078 | 292.8051198 |
| rs2284553 | G | A | 0.103192 | 0.5904 | 0.0123247 | 51874 | 5.63E-17 | 0.00515025 | 268.5368252 |
| rs2395022 | C | A | -0.177152 | 0.95885 | 0.0281553 | 51874 | 3.13E-10 | 0.002476524 | 128.7812083 |
| rs2413583 | T | C | -0.210407 | 0.1654 | 0.0168361 | 51874 | 7.72E-36 | 0.012222618 | 641.8568279 |
| rs2476601 | G | A | 0.18506 | 0.90234 | 0.0219839 | 51874 | 3.83E-17 | 0.0060359 | 314.9954897 |
| rs2538470 | G | A | -0.0749643 | 0.6378 | 0.0122847 | 51874 | 1.05E-09 | 0.002596402 | 135.0311602 |
| rs259964 | G | A | -0.0713342 | 0.5414 | 0.0119064 | 51874 | 2.08E-09 | 0.002526841 | 131.4043259 |
| rs2641348 | G | A | -0.121336 | 0.1077 | 0.0198422 | 51874 | 9.65E-10 | 0.002829671 | 147.1972123 |
| rs26528 | C | T | 0.119845 | 0.4576 | 0.0122466 | 51874 | 1.29E-22 | 0.00712977 | 372.4912161 |
| rs2847293 | T | A | -0.166911 | 0.8404 | 0.0158476 | 51874 | 6.14E-26 | 0.007473411 | 390.5797181 |
| rs28999107 | T | G | 0.0856594 | 0.4387 | 0.0126539 | 51874 | 1.29E-11 | 0.003613622 | 188.1256182 |
| rs2974935 | T | G | 0.0755507 | 0.4948 | 0.0121937 | 51874 | 5.80E-10 | 0.002853645 | 148.4479146 |
| rs3024505 | A | G | 0.165311 | 0.1573 | 0.0159634 | 51874 | 3.95E-25 | 0.007244947 | 378.5524883 |
| rs303429 | T | C | 0.0762756 | 0.5997 | 0.0124275 | 51874 | 8.38E-10 | 0.002793321 | 145.3010372 |
| rs3129871 | C | A | -0.087922 | 0.6601 | 0.0130803 | 51874 | 1.80E-11 | 0.003468854 | 180.5627359 |
| rs3197999 | A | G | 0.155114 | 0.2812 | 0.0128775 | 51874 | 2.05E-33 | 0.009726473 | 509.4871078 |
| rs34779708 | G | T | 0.134457 | 0.3512 | 0.0123872 | 51874 | 1.90E-27 | 0.008238766 | 430.9114587 |
| rs34787213 | T | C | -0.14973 | 0.1401 | 0.0183059 | 51874 | 2.85E-16 | 0.005401741 | 281.7208742 |
| rs34804116 | A | C | -0.0939086 | 0.3867 | 0.0126747 | 51874 | 1.27E-13 | 0.004183 | 217.8920183 |
| rs35164067 | A | G | -0.143373 | 0.2039 | 0.015563 | 51874 | 3.19E-20 | 0.006673437 | 348.490172 |
| rs35320439 | C | T | 0.0840555 | 0.3104 | 0.0137546 | 51874 | 9.89E-10 | 0.003024693 | 157.3728551 |
| rs35730213 | C | G | -0.151105 | 0.2807 | 0.0138184 | 51874 | 7.84E-28 | 0.009220196 | 482.7207712 |
| rs36016881 | G | A | -0.108961 | 0.1754 | 0.0170379 | 51874 | 1.60E-10 | 0.003434354 | 178.7607455 |
| rs3776414 | G | T | 0.0888353 | 0.3756 | 0.012297 | 51874 | 5.04E-13 | 0.003701601 | 192.7228374 |
| rs3801810 | A | G | 0.105079 | 0.2337 | 0.01402 | 51874 | 6.63E-14 | 0.003954753 | 205.9554656 |
| rs438475 | A | G | 0.159076 | 0.1322 | 0.0172814 | 51874 | 3.42E-20 | 0.005806179 | 302.9370237 |
| rs4795397 | G | A | 0.132338 | 0.4713 | 0.0120311 | 51874 | 3.84E-28 | 0.008727822 | 456.7157155 |
| rs4917129 | C | T | 0.095255 | 0.5957 | 0.0124591 | 51874 | 2.08E-14 | 0.004370558 | 227.7047878 |
| rs516246 | T | C | 0.114576 | 0.4652 | 0.0123121 | 51874 | 1.33E-20 | 0.006532034 | 341.057448 |
| rs559928 | C | T | 0.099124 | 0.8128 | 0.0158237 | 51874 | 3.75E-10 | 0.002990041 | 155.5645618 |
| rs56163845 | G | A | -0.091908 | 0.3096 | 0.0134853 | 51874 | 9.40E-12 | 0.00361109 | 187.9933425 |
| rs6062496 | A | G | 0.120078 | 0.5694 | 0.0124099 | 51874 | 3.82E-22 | 0.007070471 | 369.3711252 |
| rs6074022 | T | C | -0.0963211 | 0.7497 | 0.0137745 | 51874 | 2.70E-12 | 0.00348194 | 181.2462553 |
| rs6111031 | T | C | -0.282366 | 0.1591 | 0.018121 | 51874 | 9.61E-55 | 0.021333855 | 1130.753027 |
| rs61839660 | T | C | 0.148159 | 0.08983 | 0.0203346 | 51874 | 3.19E-13 | 0.003589467 | 186.8635829 |
| rs640466 | C | T | -0.0758092 | 0.3739 | 0.0124963 | 51874 | 1.31E-09 | 0.002690748 | 139.9510422 |
| rs6456426 | A | C | -0.0991795 | 0.4984 | 0.0119962 | 51874 | 1.37E-16 | 0.004918236 | 256.3796865 |
| rs6500315 | G | A | 0.145539 | 0.7753 | 0.0146056 | 51874 | 2.18E-23 | 0.007380089 | 385.6662509 |
| rs6561151 | A | G | 0.147148 | 0.2235 | 0.0142318 | 51874 | 4.68E-25 | 0.007515497 | 392.7959212 |
| rs6651252 | C | T | -0.149029 | 0.13 | 0.0183017 | 51874 | 3.86E-16 | 0.005023821 | 261.9114501 |
| rs6702421 | T | C | 0.110194 | 0.2255 | 0.0141395 | 51874 | 6.53E-15 | 0.004241445 | 220.9493901 |
| rs6738394 | A | G | 0.0777406 | 0.4519 | 0.011991 | 51874 | 8.98E-11 | 0.002993835 | 155.7625559 |
| rs6738490 | C | T | 0.226164 | 0.5274 | 0.0120892 | 51874 | 4.26E-78 | 0.025498274 | 1357.254132 |
| rs6740462 | A | C | 0.099737 | 0.7378 | 0.0141387 | 51874 | 1.74E-12 | 0.003848699 | 200.411031 |
| rs6827756 | C | T | -0.0789645 | 0.6251 | 0.0125632 | 51874 | 3.27E-10 | 0.002922528 | 152.0417305 |
| rs6908425 | C | T | 0.103988 | 0.7843 | 0.0150464 | 51874 | 4.81E-12 | 0.003658717 | 190.4818825 |
| rs7015630 | C | T | -0.0842498 | 0.2657 | 0.0137523 | 51874 | 9.00E-10 | 0.002769701 | 144.0689408 |
| rs714027 | G | A | -0.0894647 | 0.5461 | 0.0123006 | 51874 | 3.51E-13 | 0.003967946 | 206.6452625 |
| rs71624119 | A | G | -0.0923221 | 0.2422 | 0.0149485 | 51874 | 6.57E-10 | 0.003128744 | 162.8036001 |
| rs7194886 | T | C | -0.226986 | 0.4357 | 0.0121749 | 51874 | 1.42E-77 | 0.025335282 | 1348.352663 |
| rs7236492 | T | C | -0.0996611 | 0.1537 | 0.017342 | 51874 | 9.09E-09 | 0.002583923 | 134.3804793 |
| rs72727394 | T | C | 0.103233 | 0.2007 | 0.0149661 | 51874 | 5.28E-12 | 0.003419198 | 177.9691615 |
| rs727563 | T | C | -0.0921252 | 0.7973 | 0.014461 | 51874 | 1.88E-10 | 0.002743231 | 142.6883107 |
| rs7438704 | G | A | 0.0839019 | 0.6435 | 0.0126601 | 51874 | 3.42E-11 | 0.003229845 | 168.0813934 |
| rs7517847 | G | T | -0.335814 | 0.4352 | 0.0124758 | 51874 | 1.38E-159 | 0.055438461 | 3044.48544 |
| rs7608910 | G | A | 0.120609 | 0.3909 | 0.0121405 | 51874 | 2.95E-23 | 0.006926976 | 361.8224478 |
| rs76546301 | A | G | 0.225343 | 0.01822 | 0.0392973 | 51874 | 9.79E-09 | 0.001816689 | 94.40682271 |
| rs76906269 | G | A | 0.394341 | 0.01883 | 0.0370279 | 51874 | 1.75E-26 | 0.005746037 | 299.7810014 |
| rs7742214 | A | C | -0.0866616 | 0.2496 | 0.0143212 | 51874 | 1.44E-09 | 0.002813331 | 146.3448141 |
| rs7773324 | A | G | 0.078744 | 0.6002 | 0.012909 | 51874 | 1.06E-09 | 0.0029758 | 154.8214085 |
| rs7786444 | T | C | 0.112085 | 0.1163 | 0.0183382 | 51874 | 9.83E-10 | 0.002582317 | 134.2967452 |
| rs77981966 | T | C | 0.182656 | 0.07271 | 0.0222451 | 51874 | 2.19E-16 | 0.004498913 | 234.4222645 |
| rs780094 | C | T | -0.116483 | 0.6051 | 0.0120611 | 51874 | 4.56E-22 | 0.006484394 | 338.5537874 |
| rs7848647 | C | T | 0.141308 | 0.6746 | 0.0129961 | 51874 | 1.55E-27 | 0.008766523 | 458.7588048 |
| rs7969592 | G | A | -0.0731898 | 0.4746 | 0.011991 | 51874 | 1.04E-09 | 0.002671461 | 138.9452375 |
| rs9264942 | C | T | 0.150794 | 0.3529 | 0.0128298 | 51874 | 6.78E-32 | 0.010385351 | 544.362318 |
| rs9457247 | T | C | 0.123663 | 0.5398 | 0.0124045 | 51874 | 2.08E-23 | 0.007597821 | 397.1314946 |
| rs9491892 | G | T | 0.137869 | 0.1496 | 0.0163762 | 51874 | 3.80E-17 | 0.004836354 | 252.0905597 |
| rs9554587 | G | A | -0.0951561 | 0.2242 | 0.0146501 | 51874 | 8.29E-11 | 0.003149841 | 163.9048324 |
| rs9594766 | A | G | -0.0735547 | 0.5294 | 0.0121434 | 51874 | 1.39E-09 | 0.002695794 | 140.2142183 |
| rs9889296 | A | G | -0.143097 | 0.2723 | 0.0137815 | 51874 | 2.96E-25 | 0.008115048 | 424.3876736 |
