# Supplementary material for: The causal effects of inflammatory bowel disease on its ocular manifestations: A Mendelian randomization study
Source: PLoS One. 2025 Mar 12;20(3):e0316437. doi: 10.1371/journal.pone.0316437 (PMC11902285; doi:10.1371/journal.pone.0316437)
Supplement: S10 Table — (DOC) [file pone.0316437.s013.doc]

**S10 Table. The general information of the chosen genetic IVs for IBD on episcleritis.**

| SNP | effect_allele.  exposure | other_allele.  exposure | beta.  exposure | eaf.  exposure | pval.  exposure | samplesize.  exposure | se.  exposure | R2 | F |
| --- | --- | --- | --- | --- | --- | --- | --- | --- | --- |
| rs1003342 | G | A | -0.0801818 | 0.5459 | 3.30E-15 | 65642 | 0.0101765 | 0.003187471 | 209.8946065 |
| rs10748781 | A | C | -0.169179 | 0.5733 | 2.28E-63 | 65642 | 0.0100678 | 0.014003206 | 932.2245957 |
| rs10758669 | A | C | -0.148762 | 0.6504 | 4.70E-48 | 65642 | 0.0102138 | 0.010063892 | 667.3096053 |
| rs10761659 | G | A | 0.153811 | 0.5399 | 4.97E-53 | 65642 | 0.0100347 | 0.011753585 | 780.681113 |
| rs10800309 | G | A | -0.132111 | 0.6578 | 6.15E-37 | 65642 | 0.010405 | 0.007857454 | 519.8479411 |
| rs10878302 | A | T | 0.112429 | 0.92855 | 5.26E-09 | 65642 | 0.0192561 | 0.001677236 | 110.2787455 |
| rs10956252 | G | C | 0.0834795 | 0.6189 | 2.26E-16 | 65642 | 0.0101714 | 0.003287374 | 216.4949239 |
| rs11152949 | G | A | 0.105057 | 0.3195 | 7.25E-23 | 65642 | 0.0106717 | 0.004799312 | 316.5460382 |
| rs11230563 | T | C | -0.081194 | 0.348 | 1.71E-14 | 65642 | 0.0105845 | 0.002991608 | 196.9583823 |
| rs11236797 | A | C | 0.150864 | 0.4444 | 9.32E-52 | 65642 | 0.00996698 | 0.011239255 | 746.1306422 |
| rs11641016 | G | C | -0.111288 | 0.1973 | 9.51E-17 | 65642 | 0.0133908 | 0.003922898 | 258.5131472 |
| rs11677953 | A | G | 0.0790564 | 0.3969 | 2.92E-15 | 65642 | 0.0100145 | 0.002992089 | 196.9901264 |
| rs11691685 | G | A | -0.122467 | 0.08024 | 7.27E-11 | 65642 | 0.0187977 | 0.002213776 | 145.6346315 |
| rs11713774 | C | T | 0.0942858 | 0.1427 | 3.92E-11 | 65642 | 0.0142704 | 0.002175101 | 143.0848355 |
| rs11793497 | G | A | 0.156206 | 0.423 | 1.71E-54 | 65642 | 0.0100484 | 0.011910818 | 791.2505541 |
| rs1182188 | C | T | -0.0659028 | 0.2989 | 1.08E-09 | 65642 | 0.0108086 | 0.001820303 | 119.7025636 |
| rs12318183 | A | C | 0.109531 | 0.3854 | 1.67E-27 | 65642 | 0.01008 | 0.005683402 | 375.1908605 |
| rs12411259 | A | G | 0.0669054 | 0.2401 | 6.18E-09 | 65642 | 0.0115117 | 0.001633432 | 107.3938683 |
| rs1250566 | A | G | -0.100894 | 0.3161 | 4.77E-20 | 65642 | 0.0110038 | 0.004401268 | 290.1763497 |
| rs12585310 | A | G | 0.0706485 | 0.3136 | 5.25E-11 | 65642 | 0.0107636 | 0.002148766 | 141.3487555 |
| rs1267499 | C | T | 0.0821443 | 0.81 | 5.22E-11 | 65642 | 0.0125131 | 0.002076938 | 136.6139331 |
| rs12722515 | A | C | -0.0989022 | 0.1627 | 4.57E-12 | 65642 | 0.0142955 | 0.002665082 | 175.4034342 |
| rs12796489 | A | C | -0.760367 | 0.02286 | 2.87E-69 | 65642 | 0.0432241 | 0.025829115 | 1740.3755 |
| rs1292053 | G | A | 0.0701035 | 0.442 | 9.89E-13 | 65642 | 0.00982936 | 0.002424186 | 159.5102249 |
| rs1297258 | T | C | -0.114524 | 0.4249 | 5.38E-30 | 65642 | 0.0100654 | 0.006409927 | 423.4619955 |
| rs13107612 | T | C | 0.0732561 | 0.2969 | 1.62E-11 | 65642 | 0.0108741 | 0.0022405 | 147.3966387 |
| rs13204742 | T | G | 0.0916208 | 0.1267 | 5.39E-10 | 65642 | 0.0147604 | 0.001857626 | 122.1614866 |
| rs13407913 | G | A | 0.0917072 | 0.4306 | 1.69E-20 | 65642 | 0.00988181 | 0.004124092 | 271.8264403 |
| rs1363907 | A | G | 0.0815026 | 0.4212 | 4.87E-15 | 65642 | 0.0104086 | 0.003238842 | 213.2884184 |
| rs1388585 | A | G | -0.30489 | 0.98081 | 6.85E-22 | 65642 | 0.031707 | 0.00349926 | 230.4980008 |
| rs1420098 | C | T | -0.0952951 | 0.3877 | 1.83E-20 | 65642 | 0.0102776 | 0.004311528 | 284.234172 |
| rs1517352 | C | A | 0.0778816 | 0.6048 | 3.87E-14 | 65642 | 0.0102944 | 0.002899536 | 190.8789767 |
| rs1569328 | T | C | -0.0809722 | 0.1702 | 3.21E-09 | 65642 | 0.0136766 | 0.001851974 | 121.7891166 |
| rs17293632 | T | C | 0.107165 | 0.2364 | 2.71E-20 | 65642 | 0.0116106 | 0.004146191 | 273.2890984 |
| rs17694108 | A | G | 0.0857629 | 0.2797 | 1.21E-14 | 65642 | 0.0111165 | 0.002963704 | 195.1157808 |
| rs17780256 | C | A | -0.083427 | 0.1927 | 3.19E-11 | 65642 | 0.0125693 | 0.002165509 | 142.4524677 |
| rs181826 | A | C | 0.0820444 | 0.6266 | 4.05E-15 | 65642 | 0.0104471 | 0.00314987 | 207.4107629 |
| rs1847472 | A | C | -0.0672805 | 0.3421 | 6.63E-10 | 65642 | 0.0108961 | 0.002037611 | 134.0219015 |
| rs1990760 | T | C | -0.0671151 | 0.6085 | 3.56E-10 | 65642 | 0.0107006 | 0.002146164 | 141.177169 |
| rs2024092 | A | G | 0.106813 | 0.2162 | 1.12E-18 | 65642 | 0.0121076 | 0.003866688 | 254.7946351 |
| rs2050392 | A | G | 0.0691178 | 0.6003 | 1.87E-11 | 65642 | 0.0102921 | 0.002292516 | 150.8264961 |
| rs2143178 | C | T | -0.176684 | 0.1658 | 4.80E-38 | 65642 | 0.0137017 | 0.008635334 | 571.7606854 |
| rs2153283 | A | C | -0.0859637 | 0.217 | 1.54E-11 | 65642 | 0.0127458 | 0.002511202 | 165.2502924 |
| rs2266961 | G | C | -0.0906694 | 0.8035 | 2.56E-13 | 65642 | 0.0123939 | 0.002595971 | 170.843071 |
| rs2270395 | T | C | 0.0778411 | 0.7616 | 5.17E-11 | 65642 | 0.0118554 | 0.002200296 | 144.7459139 |
| rs2274351 | T | C | 0.0604993 | 0.5373 | 6.93E-09 | 65642 | 0.0104442 | 0.001819898 | 119.6758992 |
| rs2297559 | A | G | 0.0741659 | 0.6822 | 1.88E-11 | 65642 | 0.0110454 | 0.002385087 | 156.9313771 |
| rs2328546 | C | T | 0.0940162 | 0.8014 | 1.30E-13 | 65642 | 0.0126938 | 0.00281361 | 185.2064875 |
| rs2395022 | C | A | -0.181635 | 0.95885 | 8.27E-15 | 65642 | 0.0233963 | 0.002603452 | 171.3366597 |
| rs2488397 | C | G | 0.09881 | 0.2037 | 4.55E-16 | 65642 | 0.0121644 | 0.003167375 | 208.5671315 |
| rs2497318 | T | C | -0.0635464 | 0.4504 | 1.36E-10 | 65642 | 0.00989735 | 0.001999204 | 131.4905949 |
| rs2538470 | G | A | -0.0675599 | 0.6378 | 3.00E-11 | 65642 | 0.0101646 | 0.002108827 | 138.715932 |
| rs259964 | G | A | -0.0674584 | 0.5414 | 6.93E-12 | 65642 | 0.00983489 | 0.002259719 | 148.6638707 |
| rs2688608 | T | G | 0.062403 | 0.557 | 2.75E-10 | 65642 | 0.00988642 | 0.001921763 | 126.3874178 |
| rs272882 | T | G | 0.166117 | 0.6733 | 1.47E-52 | 65642 | 0.0108875 | 0.012139922 | 806.657249 |
| rs2836883 | A | G | -0.168413 | 0.2728 | 3.38E-48 | 65642 | 0.0115451 | 0.011253289 | 747.0728894 |
| rs2847278 | T | C | -0.144528 | 0.841 | 8.33E-28 | 65642 | 0.0132236 | 0.005586337 | 368.7470809 |
| rs2974935 | T | G | 0.0687256 | 0.4948 | 8.87E-12 | 65642 | 0.0100715 | 0.002361349 | 155.365796 |
| rs3024493 | A | C | 0.196922 | 0.1572 | 1.65E-50 | 65642 | 0.0131753 | 0.010275324 | 681.4746644 |
| rs3184504 | C | T | -0.0600317 | 0.5074 | 1.29E-09 | 65642 | 0.00989211 | 0.001801508 | 118.4643874 |
| rs34779708 | G | T | 0.106679 | 0.3512 | 2.07E-25 | 65642 | 0.0102408 | 0.005186247 | 342.2000022 |
| rs34856868 | A | G | -0.195275 | 0.03159 | 9.80E-09 | 65642 | 0.0340546 | 0.002333094 | 153.5024056 |
| rs35164067 | A | G | -0.11754 | 0.2039 | 2.66E-20 | 65642 | 0.0127318 | 0.004485245 | 295.7379713 |
| rs35256947 | C | T | 0.0821647 | 0.2594 | 3.87E-13 | 65642 | 0.0113176 | 0.002593906 | 170.7067829 |
| rs35730213 | C | G | -0.159624 | 0.2807 | 8.33E-45 | 65642 | 0.0113656 | 0.010289135 | 682.4001002 |
| rs36048684 | A | T | -0.0941465 | 0.1107 | 3.70E-09 | 65642 | 0.0159649 | 0.001745156 | 114.7523039 |
| rs367569 | T | C | -0.0958166 | 0.2891 | 1.93E-17 | 65642 | 0.011275 | 0.003773706 | 248.6444021 |
| rs3776414 | G | T | 0.0773716 | 0.3756 | 2.65E-14 | 65642 | 0.0101613 | 0.0028079 | 184.8295357 |
| rs3801835 | T | C | 0.064107 | 0.3447 | 1.47E-09 | 65642 | 0.0106005 | 0.001856617 | 122.0950326 |
| rs4703855 | T | C | -0.0710611 | 0.2998 | 7.16E-11 | 65642 | 0.0109034 | 0.002120057 | 139.456211 |
| rs4743820 | T | C | 0.0639523 | 0.7019 | 3.80E-09 | 65642 | 0.0108526 | 0.00171151 | 112.5361515 |
| rs4795397 | G | A | 0.138343 | 0.4713 | 8.30E-44 | 65642 | 0.00996661 | 0.009537864 | 632.0942198 |
| rs4917129 | C | T | 0.0794435 | 0.5957 | 9.48E-15 | 65642 | 0.010256 | 0.003040031 | 200.1561451 |
| rs4976646 | C | T | 0.0730113 | 0.3415 | 3.23E-12 | 65642 | 0.0104794 | 0.002397489 | 157.7493885 |
| rs516246 | T | C | 0.0755599 | 0.4652 | 1.15E-13 | 65642 | 0.0101797 | 0.002840821 | 187.0027229 |
| rs55808324 | A | G | 0.141213 | 0.09318 | 5.13E-17 | 65642 | 0.016844 | 0.003369948 | 221.9513262 |
| rs559928 | C | T | 0.094388 | 0.8128 | 3.33E-13 | 65642 | 0.0129645 | 0.002711147 | 178.4434907 |
| rs56167332 | A | C | 0.155855 | 0.3375 | 7.17E-50 | 65642 | 0.0104967 | 0.010862534 | 720.8469322 |
| rs6062496 | A | G | 0.123216 | 0.5694 | 2.11E-33 | 65642 | 0.0102312 | 0.007444846 | 492.3450993 |
| rs6074022 | T | C | -0.0742587 | 0.7497 | 8.32E-11 | 65642 | 0.0114338 | 0.002069536 | 136.126078 |
| rs6111031 | T | C | -0.264091 | 0.1591 | 1.23E-71 | 65642 | 0.0147557 | 0.018661723 | 1248.249967 |
| rs62037363 | C | T | 0.0987549 | 0.3911 | 6.36E-22 | 65642 | 0.0102618 | 0.004644951 | 306.3173817 |
| rs6456426 | A | C | -0.0643405 | 0.4984 | 8.18E-11 | 65642 | 0.0099028 | 0.002069829 | 136.1453584 |
| rs6466198 | T | A | 0.0841312 | 0.386 | 2.18E-16 | 65642 | 0.0102451 | 0.003355057 | 220.9672665 |
| rs648541 | G | A | -0.0648616 | 0.3409 | 1.22E-09 | 65642 | 0.0106716 | 0.00189053 | 124.3294522 |
| rs6500315 | G | A | 0.0766082 | 0.7753 | 1.12E-10 | 65642 | 0.0118778 | 0.002044812 | 134.4964745 |
| rs6561151 | A | G | 0.1 | 0.2235 | 3.53E-17 | 65642 | 0.0118661 | 0.003470955 | 228.6270404 |
| rs6588248 | G | T | 0.0819867 | 0.5297 | 1.38E-16 | 65642 | 0.0099185 | 0.003349051 | 220.5704085 |
| rs6651252 | C | T | -0.0908484 | 0.13 | 9.08E-10 | 65642 | 0.014833 | 0.001866926 | 122.7742508 |
| rs6708373 | G | A | 0.134178 | 0.5277 | 1.43E-41 | 65642 | 0.0099341 | 0.00897424 | 594.4034106 |
| rs6740462 | A | C | 0.0799597 | 0.7378 | 5.59E-12 | 65642 | 0.0116057 | 0.002473681 | 162.7750567 |
| rs6745185 | G | T | 0.0698267 | 0.7389 | 1.37E-09 | 65642 | 0.0115253 | 0.001881333 | 123.7234336 |
| rs67643815 | T | G | -0.0629092 | 0.5342 | 6.42E-10 | 65642 | 0.0101798 | 0.001969526 | 129.5347998 |
| rs6933404 | C | T | 0.0957518 | 0.2108 | 5.84E-15 | 65642 | 0.0122643 | 0.003050574 | 200.8523962 |
| rs71593329 | G | T | -0.0978042 | 0.1998 | 1.19E-14 | 65642 | 0.0126731 | 0.003058715 | 201.3900585 |
| rs7194886 | T | C | -0.126026 | 0.4357 | 2.53E-36 | 65642 | 0.0100135 | 0.007809944 | 516.6799543 |
| rs7240004 | G | A | -0.0665215 | 0.3795 | 1.01E-10 | 65642 | 0.0102898 | 0.002084048 | 137.0825694 |
| rs7253253 | T | G | -0.134424 | 0.95432 | 6.19E-09 | 65642 | 0.02313 | 0.001575447 | 103.5755063 |
| rs72634258 | C | T | -0.126877 | 0.1757 | 1.25E-19 | 65642 | 0.0139965 | 0.004662865 | 307.5043222 |
| rs744166 | G | A | -0.100017 | 0.4204 | 1.14E-22 | 65642 | 0.0102076 | 0.004874934 | 321.5582334 |
| rs7523442 | T | C | 0.124537 | 0.5356 | 2.76E-36 | 65642 | 0.00990061 | 0.00771542 | 510.3779514 |
| rs7547569 | C | T | -0.6472 | 0.06675 | 1.65E-170 | 65642 | 0.0232516 | 0.052186273 | 3614.114102 |
| rs7608910 | G | A | 0.126444 | 0.3909 | 2.60E-36 | 65642 | 0.0100484 | 0.007613436 | 503.5799317 |
| rs7657746 | G | A | -0.0868542 | 0.244 | 1.83E-13 | 65642 | 0.0118004 | 0.002783064 | 183.1901817 |
| rs769177 | T | C | 0.260903 | 0.02548 | 6.53E-20 | 65642 | 0.0285603 | 0.00338048 | 222.6473368 |
| rs7773324 | A | G | 0.061818 | 0.6002 | 5.84E-09 | 65642 | 0.0106192 | 0.001833997 | 120.6047674 |
| rs780094 | C | T | -0.0783055 | 0.6051 | 3.88E-15 | 65642 | 0.00996426 | 0.002930413 | 192.9176305 |
| rs7848647 | C | T | 0.13239 | 0.6746 | 3.16E-35 | 65642 | 0.0106897 | 0.007694922 | 509.0115115 |
| rs78487399 | C | G | -0.132093 | 0.90326 | 7.71E-16 | 65642 | 0.0163913 | 0.003049358 | 200.7721083 |
| rs913678 | C | T | -0.0691643 | 0.3293 | 5.35E-11 | 65642 | 0.0105418 | 0.002113071 | 138.9956608 |
| rs9264942 | C | T | 0.094692 | 0.3529 | 1.55E-18 | 65642 | 0.0107773 | 0.004095243 | 269.9170993 |
| rs9273363 | A | C | -0.193129 | 0.2748 | 3.30E-58 | 65642 | 0.0120075 | 0.014866184 | 990.541894 |
| rs941823 | C | T | 0.0830172 | 0.7509 | 6.19E-13 | 65642 | 0.0115361 | 0.002578232 | 169.6726022 |
| rs9457247 | T | C | 0.089151 | 0.5398 | 2.48E-18 | 65642 | 0.0102086 | 0.003948771 | 260.2248847 |
| rs9557207 | G | A | -0.0878448 | 0.2231 | 3.52E-13 | 65642 | 0.0120782 | 0.002675019 | 176.0591817 |
| rs974801 | G | A | -0.0727718 | 0.3799 | 7.07E-13 | 65642 | 0.0101382 | 0.002495096 | 164.1877638 |
| rs9836291 | A | G | 0.160867 | 0.2878 | 9.61E-53 | 65642 | 0.0105244 | 0.010608566 | 703.8127118 |
| rs9889296 | A | G | -0.104999 | 0.2723 | 1.35E-20 | 65642 | 0.0112854 | 0.004369184 | 288.0517911 |
