# Supplementary material for: The causal effects of inflammatory bowel disease on its ocular manifestations: A Mendelian randomization study
Source: PLoS One. 2025 Mar 12;20(3):e0316437. doi: 10.1371/journal.pone.0316437 (PMC11902285; doi:10.1371/journal.pone.0316437)
Supplement: S18 Table — (DOC) [file pone.0316437.s021.doc]

**S18 Table. The general information of the chosen genetic IVs for UC on corneal disease.**

| SNP | effect_allele.  exposure | other_allele.  exposure | beta.  exposure | eaf.  exposure | samplesize.  exposure | pval.  exposure | se.  exposure | R2 | F |
| --- | --- | --- | --- | --- | --- | --- | --- | --- | --- |
| rs10185424 | G | T | -0.0965995 | 0.5396 | 47745 | 1.47E-14 | 0.0125621 | 0.004636465 | 222.3898631 |
| rs10748783 | A | C | -0.164756 | 0.5237 | 47745 | 7.73E-39 | 0.0126394 | 0.013541776 | 655.4003022 |
| rs10758669 | A | C | -0.143218 | 0.6504 | 47745 | 1.04E-28 | 0.0128828 | 0.009327756 | 449.5281272 |
| rs10761659 | G | A | 0.117315 | 0.5399 | 47745 | 1.50E-20 | 0.0126238 | 0.006837584 | 328.6942258 |
| rs1077773 | A | G | 0.0721282 | 0.5238 | 47745 | 5.96E-09 | 0.012398 | 0.002595345 | 124.2319733 |
| rs10910092 | G | A | -0.0863506 | 0.4676 | 47745 | 1.42E-11 | 0.0127821 | 0.003712558 | 177.9091616 |
| rs11150589 | C | T | -0.0798521 | 0.5268 | 47745 | 3.28E-10 | 0.0127057 | 0.003179019 | 152.2599619 |
| rs111830527 | A | G | -0.192324 | 0.05254 | 47745 | 5.09E-11 | 0.0292804 | 0.003682544 | 176.4655278 |
| rs11641184 | A | C | 0.0780232 | 0.4762 | 47745 | 4.24E-10 | 0.0124939 | 0.003036913 | 145.4330204 |
| rs11676348 | T | C | 0.0744362 | 0.4763 | 47745 | 2.08E-09 | 0.0124232 | 0.00276415 | 132.3345855 |
| rs1182188 | C | T | -0.107615 | 0.2989 | 47745 | 5.03E-15 | 0.0137507 | 0.004853796 | 232.8650503 |
| rs12103 | C | T | -0.0995588 | 0.8166 | 47745 | 9.96E-10 | 0.0162944 | 0.002968917 | 142.1670711 |
| rs12132349 | A | T | -0.166942 | 0.2809 | 47745 | 3.64E-31 | 0.0143783 | 0.011259063 | 543.6625743 |
| rs12318183 | A | C | 0.162178 | 0.3854 | 47745 | 1.44E-37 | 0.0126604 | 0.012460003 | 602.383619 |
| rs12720356 | C | A | 0.153279 | 0.08572 | 47745 | 1.67E-11 | 0.0227683 | 0.003682618 | 176.4691088 |
| rs12796489 | A | C | -0.67649 | 0.02286 | 47745 | 1.22E-33 | 0.0559623 | 0.020444937 | 996.4755044 |
| rs1297256 | T | C | -0.10106 | 0.4249 | 47745 | 2.10E-15 | 0.0127351 | 0.004991358 | 239.4978032 |
| rs13136827 | C | T | -0.111814 | 0.1622 | 47745 | 2.35E-10 | 0.0176452 | 0.003397923 | 162.780166 |
| rs17694108 | A | G | 0.0958444 | 0.2797 | 47745 | 6.17E-12 | 0.0139396 | 0.003701428 | 177.3738367 |
| rs17780256 | C | A | -0.115389 | 0.1927 | 47745 | 6.13E-13 | 0.0160316 | 0.004142624 | 198.6040235 |
| rs1801274 | G | A | -0.170896 | 0.4958 | 47745 | 1.43E-41 | 0.012653 | 0.014601691 | 707.4586276 |
| rs1990760 | T | C | -0.0855732 | 0.6085 | 47745 | 1.78E-10 | 0.0134145 | 0.003488975 | 167.1573537 |
| rs2395022 | C | A | -0.183896 | 0.95885 | 47745 | 2.88E-10 | 0.029166 | 0.002668671 | 127.7512967 |
| rs2516440 | A | G | -0.0997745 | 0.3222 | 47745 | 4.40E-13 | 0.0137762 | 0.004348067 | 208.4963134 |
| rs272882 | T | G | 0.145856 | 0.6733 | 47745 | 6.67E-26 | 0.0138588 | 0.009359149 | 451.0553255 |
| rs2836883 | A | G | -0.227134 | 0.2728 | 47745 | 1.47E-53 | 0.0147424 | 0.020468807 | 997.6632269 |
| rs3024493 | A | C | 0.226278 | 0.1572 | 47745 | 1.42E-43 | 0.0163471 | 0.013567247 | 656.6499996 |
| rs34659678 | T | C | 0.209947 | 0.05734 | 47745 | 5.95E-17 | 0.0250948 | 0.004764991 | 228.5841801 |
| rs35223180 | T | G | -0.141003 | 0.1791 | 47745 | 1.04E-15 | 0.0175766 | 0.005846185 | 280.7557532 |
| rs3774937 | C | T | 0.099276 | 0.3257 | 47745 | 4.61E-14 | 0.0131621 | 0.004329019 | 207.5789495 |
| rs4366152 | C | T | 0.120371 | 0.68 | 47745 | 7.79E-19 | 0.0135814 | 0.00630569 | 302.9629534 |
| rs4456788 | A | G | -0.102758 | 0.6106 | 47745 | 7.07E-16 | 0.0127344 | 0.005021275 | 240.9405732 |
| rs4656958 | G | A | 0.0824158 | 0.6821 | 47745 | 2.82E-09 | 0.0138702 | 0.002945707 | 141.052383 |
| rs4676410 | A | G | 0.142022 | 0.2038 | 47745 | 1.85E-19 | 0.0157426 | 0.006545873 | 314.5788236 |
| rs4728142 | A | G | 0.0969671 | 0.439 | 47745 | 1.92E-14 | 0.0126655 | 0.004631335 | 222.1426417 |
| rs4743820 | T | C | 0.0809228 | 0.7019 | 47745 | 4.05E-09 | 0.0137572 | 0.002740369 | 131.1929454 |
| rs4747886 | T | C | 0.0738479 | 0.4081 | 47745 | 9.58E-09 | 0.0128701 | 0.00263464 | 126.1178779 |
| rs4795397 | G | A | 0.139853 | 0.4713 | 47745 | 1.01E-28 | 0.012577 | 0.00974721 | 469.9416636 |
| rs4812833 | A | G | 0.103346 | 0.5188 | 47745 | 1.87E-16 | 0.0125571 | 0.005332648 | 255.9615712 |
| rs483905 | A | G | 0.0849927 | 0.289 | 47745 | 3.16E-10 | 0.0135107 | 0.002968662 | 142.154819 |
| rs4947328 | G | A | 0.239455 | 0.02366 | 47745 | 3.38E-10 | 0.038129 | 0.002649071 | 126.8105385 |
| rs4976646 | C | T | 0.0787748 | 0.3415 | 47745 | 2.52E-09 | 0.0132165 | 0.002790944 | 133.6209615 |
| rs55808324 | A | G | 0.127209 | 0.09318 | 47745 | 1.47E-09 | 0.021035 | 0.002734699 | 130.9207442 |
| rs56167332 | A | C | 0.141368 | 0.3375 | 47745 | 7.27E-27 | 0.0131737 | 0.008937003 | 430.5269344 |
| rs6062496 | A | G | 0.113898 | 0.5694 | 47745 | 9.14E-19 | 0.0128769 | 0.006361414 | 305.657416 |
| rs6111031 | T | C | -0.260863 | 0.1591 | 47745 | 1.33E-42 | 0.0190685 | 0.018208304 | 885.4414472 |
| rs61893460 | A | G | 0.121119 | 0.4447 | 47745 | 4.60E-22 | 0.0125424 | 0.007245183 | 348.4312136 |
| rs6426833 | A | G | 0.232387 | 0.536 | 47745 | 3.77E-76 | 0.0125836 | 0.026861881 | 1317.867188 |
| rs6466198 | T | A | 0.133923 | 0.386 | 47745 | 1.90E-25 | 0.0128457 | 0.008501509 | 409.3677798 |
| rs661054 | G | A | -0.124858 | 0.3408 | 47745 | 3.18E-20 | 0.0135529 | 0.007004539 | 336.776648 |
| rs6920220 | A | G | 0.146936 | 0.2086 | 47745 | 4.78E-22 | 0.015222 | 0.007128477 | 342.7783802 |
| rs7240004 | G | A | -0.0823655 | 0.3795 | 47745 | 2.50E-10 | 0.0130179 | 0.003195025 | 153.0290028 |
| rs7547569 | C | T | -0.495701 | 0.06675 | 47745 | 8.71E-65 | 0.0291648 | 0.030613914 | 1507.758472 |
| rs7608910 | G | A | 0.127099 | 0.3909 | 47745 | 1.25E-23 | 0.0126851 | 0.007692518 | 370.1109822 |
| rs76546301 | A | G | 0.26496 | 0.01822 | 47745 | 1.05E-10 | 0.0410174 | 0.002511616 | 120.213997 |
| rs76904798 | T | C | 0.104624 | 0.1368 | 47745 | 2.78E-09 | 0.0176013 | 0.002585176 | 123.7439718 |
| rs7711427 | C | A | 0.088938 | 0.613 | 47745 | 3.67E-12 | 0.0127983 | 0.003752979 | 179.8534705 |
| rs7738430 | C | T | 0.367964 | 0.02624 | 47745 | 3.51E-27 | 0.0340763 | 0.006919209 | 332.6454159 |
| rs9271255 | T | C | -0.284959 | 0.732 | 47745 | 1.31E-94 | 0.013809 | 0.031859623 | 1571.129556 |
| rs9271858 | G | A | 0.122709 | 0.511 | 47745 | 2.42E-21 | 0.0129368 | 0.007525105 | 361.9951601 |
| rs941823 | C | T | 0.108669 | 0.7509 | 47745 | 1.39E-13 | 0.0146906 | 0.00441771 | 211.8506062 |
| rs9611131 | C | T | -0.142696 | 0.1477 | 47745 | 3.84E-15 | 0.0181548 | 0.005126566 | 246.0188868 |
| rs9836291 | A | G | 0.170257 | 0.2878 | 47745 | 8.20E-38 | 0.0132458 | 0.011883181 | 574.1615708 |
| rs9891119 | C | A | -0.0895467 | 0.3536 | 47745 | 1.72E-11 | 0.0133101 | 0.003665581 | 175.6496718 |
| rs9941524 | G | A | 0.0977156 | 0.4559 | 47745 | 2.15E-14 | 0.0127881 | 0.00473703 | 227.2364411 |
