# Supplementary material for: The causal effects of inflammatory bowel disease on its ocular manifestations: A Mendelian randomization study
Source: PLoS One. 2025 Mar 12;20(3):e0316437. doi: 10.1371/journal.pone.0316437 (PMC11902285; doi:10.1371/journal.pone.0316437)
Supplement: S19 Table — (DOC) [file pone.0316437.s022.doc]

**S19 Table. Results of MR analyses.**

| Exposure | Outcome | Method | nsnp | b | se | or | P | P_FDR |
| --- | --- | --- | --- | --- | --- | --- | --- | --- |
| IBD | Iridocyclitis | IVW | 115 | 0.169 | 0.026 | 1.184 | <0.001 | <0.001 |
|  |  | MR Egger | 115 | 0.244 | 0.065 | 1.276 | <0.001 | 0.003 |
|  |  | Weighted median | 115 | 0.161 | 0.040 | 1.174 | <0.001 | 0.001 |
|  |  | Weighted mode | 115 | 0.163 | 0.069 | 1.177 | 0.020 | 0.080 |
| CD |  | IVW | 111 | 0.078 | 0.024 | 1.082 | 0.001 | 0.007 |
|  |  | MR Egger | 111 | 0.137 | 0.066 | 1.146 | 0.040 | 0.143 |
|  |  | Weighted median | 111 | 0.103 | 0.035 | 1.108 | 0.003 | 0.019 |
|  |  | Weighted mode | 111 | 0.089 | 0.050 | 1.093 | 0.076 | 0.250 |
| UC |  | IVW | 58 | 0.175 | 0.034 | 1.192 | <0.001 | <0.001 |
|  |  | MR Egger | 58 | 0.256 | 0.093 | 1.292 | 0.008 | 0.040 |
|  |  | Weighted median | 58 | 0.174 | 0.046 | 1.191 | 0.000 | 0.003 |
|  |  | Weighted mode | 58 | 0.187 | 0.076 | 1.206 | 0.016 | 0.070 |
| IBD | Uveitis | IVW | 119 | 0.120 | 0.030 | 1.128 | <0.001 | 0.001 |
|  |  | MR Egger | 119 | 0.267 | 0.073 | 1.306 | 0.000 | 0.003 |
|  |  | Weighted median | 119 | 0.109 | 0.045 | 1.115 | 0.016 | 0.070 |
|  |  | Weighted mode | 119 | 0.092 | 0.081 | 1.096 | 0.261 | 0.560 |
| CD |  | IVW | 113 | 0.074 | 0.025 | 1.077 | 0.003 | 0.019 |
|  |  | MR Egger | 113 | 0.124 | 0.069 | 1.132 | 0.073 | 0.250 |
|  |  | Weighted median | 113 | 0.102 | 0.037 | 1.107 | 0.006 | 0.035 |
|  |  | Weighted mode | 113 | 0.132 | 0.057 | 1.141 | 0.022 | 0.085 |
| UC |  | IVW | 63 | 0.142 | 0.039 | 1.153 | <0.001 | 0.003 |
|  |  | MR Egger | 63 | 0.317 | 0.098 | 1.374 | 0.002 | 0.014 |
|  |  | Weighted median | 63 | 0.123 | 0.051 | 1.131 | 0.017 | 0.070 |
|  |  | Weighted mode | 63 | 0.073 | 0.097 | 1.075 | 0.458 | 0.778 |
| IBD | Scleritis | IVW | 118 | -0.057 | 0.126 | 0.945 | 0.655 | 0.839 |
|  |  | MR Egger | 118 | -0.187 | 0.312 | 0.830 | 0.550 | 0.837 |
|  |  | Weighted median | 118 | -0.244 | 0.192 | 0.784 | 0.205 | 0.508 |
|  |  | Weighted mode | 118 | -0.500 | 0.342 | 0.607 | 0.146 | 0.439 |
| CD |  | IVW | 114 | 0.011 | 0.110 | 1.011 | 0.919 | 0.952 |
|  |  | MR Egger | 114 | 0.314 | 0.308 | 1.369 | 0.309 | 0.611 |
|  |  | Weighted median | 114 | 0.088 | 0.177 | 1.092 | 0.620 | 0.839 |
|  |  | Weighted mode | 114 | 0.039 | 0.267 | 1.040 | 0.885 | 0.952 |
| UC |  | IVW | 62 | 0.024 | 0.158 | 1.025 | 0.878 | 0.952 |
|  |  | MR Egger | 62 | -0.147 | 0.400 | 0.863 | 0.714 | 0.857 |
|  |  | Weighted median | 62 | -0.107 | 0.216 | 0.899 | 0.622 | 0.839 |
|  |  | Weighted mode | 62 | -0.140 | 0.360 | 0.870 | 0.699 | 0.854 |
| IBD | Episcleritis | IVW | 118 | -0.005 | 0.053 | 0.995 | 0.924 | 0.952 |
|  |  | MR Egger | 118 | -0.057 | 0.131 | 0.944 | 0.662 | 0.839 |
|  |  | Weighted median | 118 | -0.085 | 0.085 | 0.918 | 0.318 | 0.611 |
|  |  | Weighted mode | 118 | -0.130 | 0.131 | 0.878 | 0.322 | 0.611 |
| CD |  | IVW | 114 | -0.025 | 0.046 | 0.975 | 0.593 | 0.837 |
|  |  | MR Egger | 114 | 0.026 | 0.130 | 1.027 | 0.840 | 0.945 |
|  |  | Weighted median | 114 | -0.088 | 0.076 | 0.916 | 0.249 | 0.560 |
|  |  | Weighted mode | 114 | -0.150 | 0.116 | 0.861 | 0.199 | 0.508 |
| UC |  | IVW | 62 | 0.024 | 0.059 | 1.024 | 0.691 | 0.854 |
|  |  | MR Egger | 62 | -0.226 | 0.149 | 0.798 | 0.134 | 0.418 |
|  |  | Weighted median | 62 | -0.066 | 0.090 | 0.937 | 0.465 | 0.778 |
|  |  | Weighted mode | 62 | -0.192 | 0.170 | 0.826 | 0.265 | 0.560 |
| IBD | Optic neuritis | IVW | 118 | 0.072 | 0.065 | 1.075 | 0.265 | 0.560 |
|  |  | MR Egger | 118 | 0.095 | 0.160 | 1.100 | 0.554 | 0.837 |
|  |  | Weighted median | 118 | 0.053 | 0.098 | 1.054 | 0.590 | 0.837 |
|  |  | Weighted mode | 118 | 0.012 | 0.157 | 1.012 | 0.938 | 0.952 |
| CD |  | IVW | 113 | 0.037 | 0.055 | 1.038 | 0.497 | 0.813 |
|  |  | MR Egger | 113 | -0.090 | 0.153 | 0.914 | 0.559 | 0.837 |
|  |  | Weighted median | 113 | 0.000 | 0.080 | 1.000 | 0.997 | 0.997 |
|  |  | Weighted mode | 113 | -0.258 | 0.214 | 0.773 | 0.232 | 0.556 |
| UC |  | IVW | 61 | 0.092 | 0.066 | 1.097 | 0.163 | 0.452 |
|  |  | MR Egger | 61 | 0.021 | 0.174 | 1.022 | 0.903 | 0.952 |
|  |  | Weighted median | 61 | 0.073 | 0.098 | 1.075 | 0.459 | 0.778 |
|  |  | Weighted mode | 61 | 0.035 | 0.171 | 1.036 | 0.837 | 0.945 |
| IBD | Corneal disease | IVW | 123 | -0.168 | 0.118 | 0.846 | 0.154 | 0.443 |
|  |  | MR Egger | 123 | -0.242 | 0.278 | 0.785 | 0.386 | 0.694 |
|  |  | Weighted median | 123 | -0.039 | 0.193 | 0.961 | 0.839 | 0.945 |
|  |  | Weighted mode | 123 | -0.023 | 0.266 | 0.978 | 0.932 | 0.952 |
| CD |  | IVW | 117 | -0.065 | 0.102 | 0.937 | 0.528 | 0.837 |
|  |  | MR Egger | 117 | 0.074 | 0.278 | 1.077 | 0.791 | 0.933 |
|  |  | Weighted median | 117 | -0.169 | 0.161 | 0.844 | 0.293 | 0.603 |
|  |  | Weighted mode | 117 | -0.108 | 0.249 | 0.897 | 0.664 | 0.839 |
| UC |  | IVW | 65 | -0.128 | 0.132 | 0.880 | 0.333 | 0.615 |
|  |  | MR Egger | 65 | -0.433 | 0.325 | 0.649 | 0.188 | 0.502 |
|  |  | Weighted median | 65 | -0.104 | 0.194 | 0.901 | 0.590 | 0.837 |
|  |  | Weighted mode | 65 | -0.138 | 0.290 | 0.871 | 0.635 | 0.839 |
